# Unveiling the Crucial Role of Chemical Enhancement in the SERS Analysis of Amphetamine–Metal Interactions on Gold and Silver Surfaces: Importance of Selective Amplification of the Narrow Interval of Vibrational Modes

**DOI:** 10.1021/acs.analchem.3c05189

**Published:** 2024-03-07

**Authors:** Valerie Smeliková, Ivan Kopal, Martin Člupek, Marcela Dendisová, Marie Švecová

**Affiliations:** † Department of Physical Chemistry, University of Chemistry and Technology Prague, Technická 5, 166 28 Prague 6, Czech Republic; ‡ Department of Analytical Chemistry, University of Chemistry and Technology Prague, Technická 5, 166 28 Prague 6, Czech Republic

## Abstract

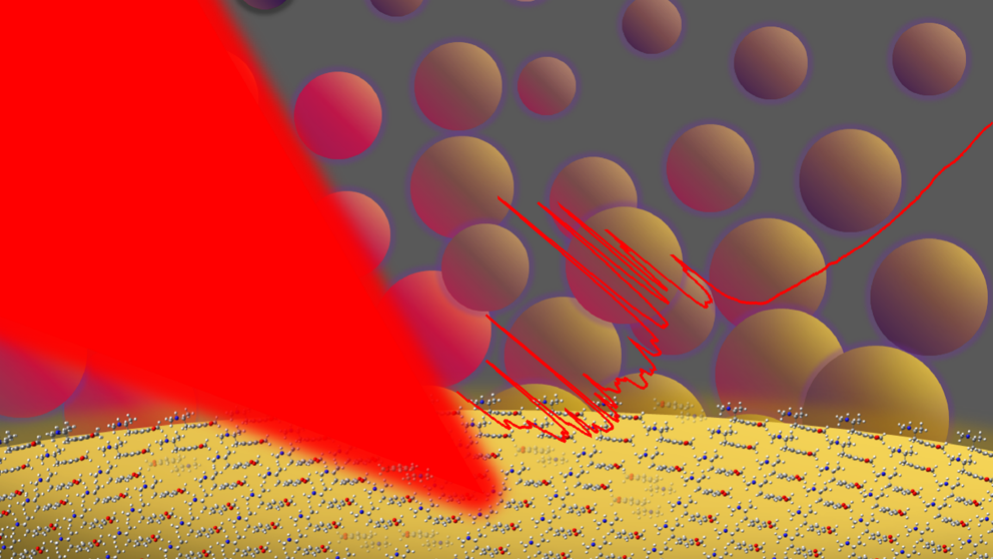

The use of addictive substances, including drugs, poses significant health risks and contributes to various social problems, such as increased crime rates associated with substance-induced aggressive behavior. To address these challenges, possession of addictive substances is legally prohibited. However, detecting and analyzing these substances remain a complex task for law enforcement, primarily due to the presence of adulterants or limited sample quantities. In response to the evolving illicit market, continuous development and adaptation of analytical techniques are essential. One approach is the utilization of surface-enhanced Raman scattering (SERS) spectroscopy, which involves adsorbing the analyte onto nanostructured plasmonic surfaces. This study explores the potential of SERS in detecting amphetamine-based addictive stimulants with a specific focus on the properties of enhancing surfaces chosen. Comparative investigations were performed using silver and gold surfaces, with gold colloidal systems demonstrating a favorable performance. Moreover, to provide a comprehensive interpretation of the measured spectra, extensive density functional theory (DFT) calculations were conducted, allowing for a deeper understanding of the observed spectral features and molecular interactions with the metal surfaces. This review presents insights into the role of chemical enhancement in SERS analysis of amphetamine–metal interactions, shedding light on the selective amplification of vibrational modes. These findings, supported by DFT calculations, have implications in the fields of spectroscopy, physical chemistry, and drug analysis, providing valuable contributions to forensic applications and a deeper understanding of chemical enhancement phenomena. We present the impact of the secondary resonances of Stokes-scattered photons. This illustrates the significance of recognizing the constraints of the frequently employed “E^4^” approximation, even in measurements involving multiple molecules rather than single molecules.

## Introduction

**Figure 1:**
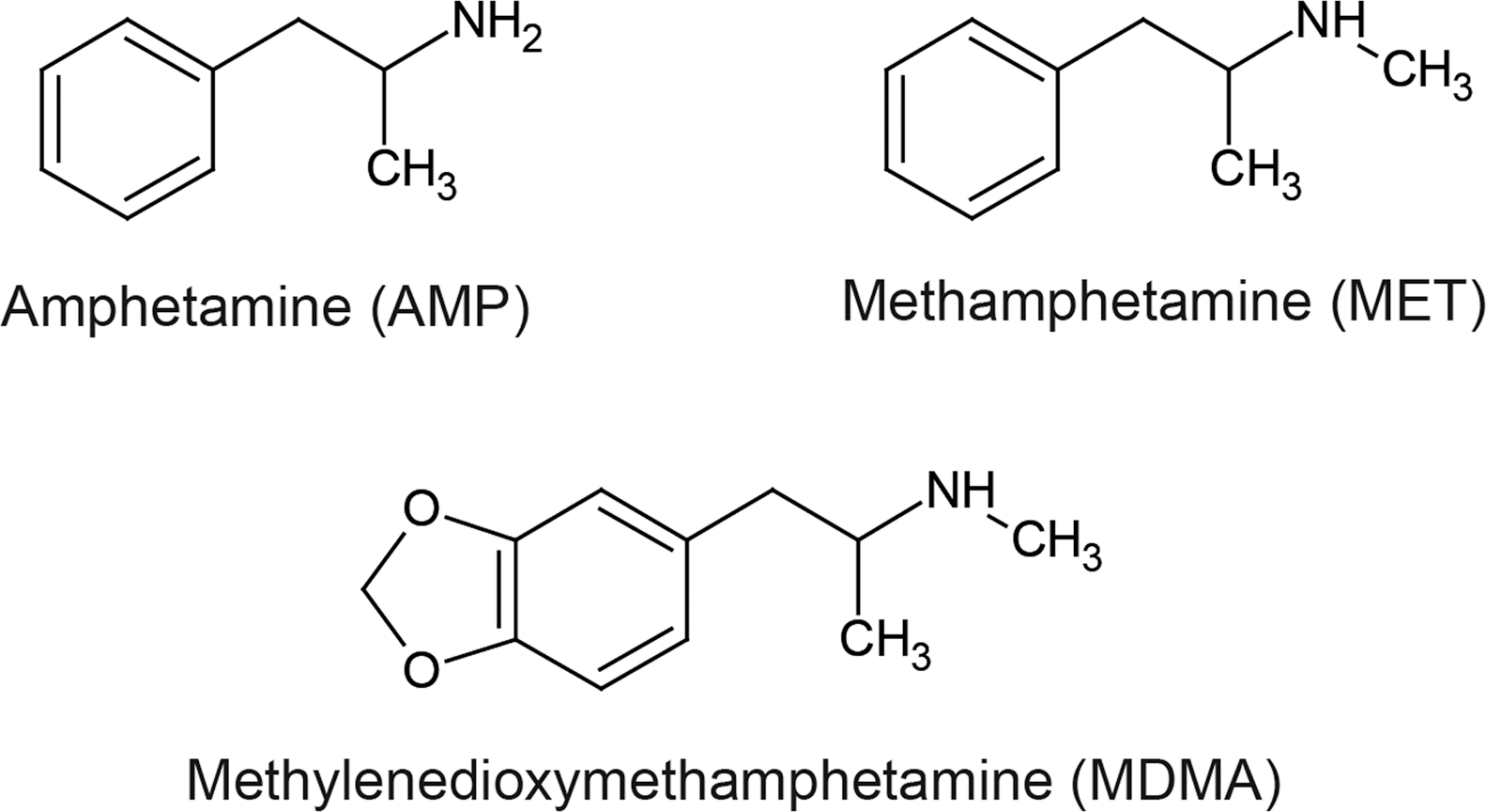
Selected amphetamine-based drugs’ structures.

In general, addictive substances or drugs are known to affect the body’s natural processes through specific interactions with receptors, enzymes, or neurotransmitters, resulting in changes in mood, perception, enjoyment, and behavior. The classification of addictive substances is based on the Czech Code: Sec. Two Paragraph 1 Lit. (a) of the Act No. 167/1998 Coll., on addictive substances, which defines them as “substances capable of adversely affecting an individual’s psyche, control, recognition abilities, or social behaviour”.^1^ The concept of an addictive substance is known to the legal system in other legal regulations as well (e.g., Paragraph 130 of the Act No. 40/2009 Coll. of Penal Code). One group of drugs are those based on amphetamine, for example, amphetamine (AMP), methamphetamine (MET), and methylenedioxymethamphetamine (MDMA). Although these substances (Figure 1) also have potential in the field of medicine, they currently find “applications” mainly as a stimulant substance, the use of which leads very quickly to the development of a hard-to-treat addiction.^2,3^

Therefore, they pose significant risks to society and are included in the list of prohibited substances in criminal codes. Detecting addictive substances is thus a crucial task for law enforcement and forensic laboratories. The emphasis lies in the analysis of trace amounts of substances and their additives deliberately introduced by manufacturers or distributors to hinder identification.

Raman spectroscopy, a powerful analytical technique, can be utilized for the identification and detection of addictive substances.^4^ However, the naturally low quantum yields of this phenomenon make this technique difficult to apply for monitoring lower concentrations of the studied substances. To overcome this problem, it is directly proposed to use the phenomenon of surface-enhanced Raman scattering (SERS), which, as a result of the interaction of the monitored molecules with the surfaces of plasmonic structures, enables several orders of magnitude amplification of the observed signal.^5,6^ Although this technique is considered already explored and nonapplicable in practice because of the challenging interpretation of obtained spectra,^7^ it has become the workhorse of many research teams, not only in the fields of physical research but also in the areas of applications in analytical chemistry.^8−11^ The SERS phenomena complexity is accompanied by a variety of experimental factors, such as, the enhancing substrate used,^8,12^ age of the substrate,^13^ the type of molecular interaction with the substrate,^12^ or the selected excitation wavelength,^8,14^ which may affect resulting profiles of observed spectra.^15^ In the future, it also offers an ever-increasing potential for the study of single molecules and combinations with tip-related techniques, although the complexity of the phenomena could be even more remarkable in the level of single molecules.^16−21^ In short, it is generally considered that the remarkable enhancement in SERS spectra arises from two complementary mechanisms, electromagnetic enhancement and chemical enhancement.^5,6^ The electromagnetic enhancement is attributed to the excitation of localized surface plasmons in the metal nanoparticles, which results in a generation of intense electromagnetic fields near the surface, enhancing the Raman scattering signals of the adsorbed molecules.^22−24^ It is usually believed that the selection of the enhancing substrate mostly affects the operation of electromagnetic mechanisms, especially because of different physical properties of the prepared nanostructures (Fermi energy, permittivity, etc.) and different morphologies, resulting in different hot-spot distributions or levels of antenna effect.^5,6,23^ The chemical enhancement, on the other hand, stems from the specific interactions between the analyte molecules and the metal surface, leading to changes in the molecular polarizability and charge transfer effects.^22,25,26^ Not exceptionally, the chemical enhancement effects are concentration-dependent, for example, because of the different manners of molecules respective to the number and types of available binding sites on the substrate surface.^27^ These interactions can result in alterations of the vibrational modes and the intensity of the Raman bands, providing unique spectral signatures that can be utilized for the identification and quantification of addictive substances, even at trace levels.^5,6,22^

In this work, we demonstrate the possibility of SERS applications for the study of amphetamine-based addictive substances. Although this possibility has already been investigated before,^28−30^ in this work, we present the opportunity of using easily prepared amplifying substrates based on colloidal solutions^31,32^ to not only detect such addictive substances but also to clarify their adsorption behavior in the presence of Ag and Au nanoparticles and to explain changes in the spectral response after interaction with the nanoparticles. Furthermore, we manifest the occurrence of an ongoing chemical mechanism of enhancement, which, aside from the additional signal enhancement, notably improves the specificity of the presented methodology. We supplemented the experimental data with a detailed interpretation using complex density functional theory (DFT) calculations, considering the possibility of molecule–metal complex genesis. For the measurement of SERS spectra, we chose an instrument with an excitation wavelength of 785 nm, which is the most common wavelength of portable Raman spectrometers, which, in combination with easily prepared substrates, creates the presented results easily translatable, for example, into common forensic and clinical practice. In addition to purely practical aspects, this work also deals with physicochemical effects that can be observed in the mentioned spectra and that have an unquestionable impact on the obtained experimental data. The secondary resonation of Stokes-scattered photons with a molecule–metal complex energy effect is presented. This illustrates the significance of recognizing the constraints of the frequently employed “E^4^” approximation even in the nonsingle molecule measurements.

## Experimental Section

### Materials

Amphetamine sulfate (≥99.00%), methylenedioxymethamphetamine chloride (≥99.00%), and methamphetamine chloride (≥99.00%) were obtained from the Laboratory of Forensic Analysis of Biologically Active Substances, UCT, Prague. Silver nitrate (≥99.00%), hydroxylamine hydrochloride (HA·HCl, 99.00%), and tetrachloroauric acid (≥99.00%) were purchased from Sigma-Aldrich (Czech Republic). Sodium hydroxide was obtained from Penta (Czech Republic).

### Colloid Preparation

In this work, two types of colloids were prepared. Both of them were in the form of hydrosols, where Milli-Q water was used as a solvent. Because of the high stability of the hydrosols, the preparation procedures were ongoing at laboratory temperature.

Silver colloidal solutions of nanoparticles (AgNPs) were prepared according to the modified preparation procedure by authors Leopold and Lendl.^31^ Gold colloidal solutions of nanoparticles (AuNPs) were also prepared on the principle of hydroxylamine reduction according to Tódor et al.^32^ The description of both preparation procedures in detail is given in the Supporting Information (SI), which is enriched by the calculation of nanoparticles’ concentrations in solutions (Tables S1 and S2).

Immediately after the preparation of the colloids, the aqueous solution of the studied substances was added to the colloids to achieve final concentrations of 10^–3^, 10^–4^, and 10^–5^ mol/L. The mixtures of the colloids with analytes were characterized immediately after mixing.

### Raman and SERS Spectroscopy

SERS spectra were measured using a dispersive spectrometer iRaman Plus (B&W Tek), which is equipped with a diode laser emitting radiation with a wavelength of 785 nm as a radiation source with a maximum power of 350 mW (per sample after passing the radiation through fiber optics). The measuring range of the instrument is from 65 cm^–1^ up to 3500 cm^–1^ and its medium resolution is better than 4.5 cm^–1^. Colloidal solutions of nanoparticles were measured in a solution sample compartment. BWSpec Software (B&W Tek) was used for the measurements themselves. The samples were recorded with the 10% power of the laser (ca. 35 mW) with an acquisition number of 10 and an exposure time of 5 s. Five spectra were recorded for each colloidal solution. The solutions were shaken between each measurement, and finally the raw data were averaged in an OMNIC program (Thermo Fisher Scientific).

Another system for SERS measurements which was used was a MultiRAM FT Raman spectrometer (Bruker, USA and Germany), where the source of the excitation wavelength is a solid Nd:YAG laser (1064 nm), and the spectrometer is further equipped with a highly sensitive Ge detector cooled by liquid nitrogen. The instrument range is from 50 cm^–1^ up to 3600 cm^–1^. The measurement was carried out using the OPUS program (Bruker). The samples were measured with a laser power of 300 mW, a resolution of 4 cm^–1^, and a number of scans of 1024. Measurements were repeated three times with 10 s intervals. Recorded raw spectra were averaged in the already mentioned OMNIC program.

### Extinction UV–Vis Spectroscopy

The prepared NPs were characterized by UV–vis spectroscopy for which a CARY 50 single-beam spectrometer (Varian) was used. The spectral range of this instrument is 190–1100 nm; however, measurements were carried out only in the range of 200–1000 nm. The radiation source is a xenon discharge lamp operating in pulse mode, and the maximum scanning speed of the spectrometer is 360 nm/min. A cuvette with a thickness of 5 mm was chosen for the solution sampling. AuNPs had to be diluted with water in a volume ratio of 1:3 before the given measurement to avoid supersaturation of the detector. The control and recording of the spectra take place with the help of Cary WinUV software.

### DFT Calculations

All of the mentioned DFT calculations were performed with the B3LYP calculation method with the LANL2DZ basis set using Gaussian 16W software. The calculations of frequencies and UV–vis spectra were preceded in all cases by optimization of the molecular geometry. UV–vis spectra are presented unscaled. Raman spectra are scaled in the main text by a scaling factor that is 0.97 for AMP and MET and 0.98 for MDMA. In the Supporting Information, all spectra are presented unscaled.

## Results and Discussion

In the following part of the text, the SERS spectra of molecules adsorbed on the surface of AuNPs or AgNPs (Au- or Ag-SERS spectra) are presented. Except for the paragraph “Excitation Wavelength Dependence”, where excitation wavelengths 785 and 1064 nm were used to compare, in the rest of the text, the former one was used for all SERS measurements. The spectra shown in the figures are the average spectra from five measurements (three in the case of 1064 nm). In all cases, the spectra are shown in full-scaled view in order to highlight the changes in the spectral profiles. In contrast, the extinction spectra are displayed in common-scaled mode, but with a different axis for the experimental and calculated data, for better orientation in the data. The results of DFT calculations are displayed both for extinction spectra (for all considered molecular complexes) and SERS spectra (only for the most probable molecular complex; spectra of other complexes are included in the Supporting Information).

In the following sections, we attribute changes in the extinction spectra to the formation of molecule–metal complexes. Nevertheless, it should be noted that aggregation of nanoparticles could be partially responsible for a similar effect, i.e., forming of a secondary extinction band and shifting of the original one, although we assume that this is not the main effect causing changes in the extinction and SERS spectra. This idea can be supported by the presence of other newly formed extinction bands, which occur in the spectra of nanoparticles modified by the addition of analytes (bands around 400 nm, which can be seen in Figures 4 and 6). To the best of our knowledge, aggregation of nanoparticles causes a red shift of the surface plasmon resonance band in all cases, and thus, these bands could not be attributed to this effect. However, these newly formed, low-positioned extinction bands can be explained by our calculation and are connected to the molecule–metal complexes. Furthermore, results presented in the sections “Narrow Interval Chemical Enhancement” and “Excitation Wavelength Dependence” are practically independent of the exact origin of the newly formed band, as they relate courses of the extinction and SERS spectra.

### SERS of Amphetamine

**Figure 2:**
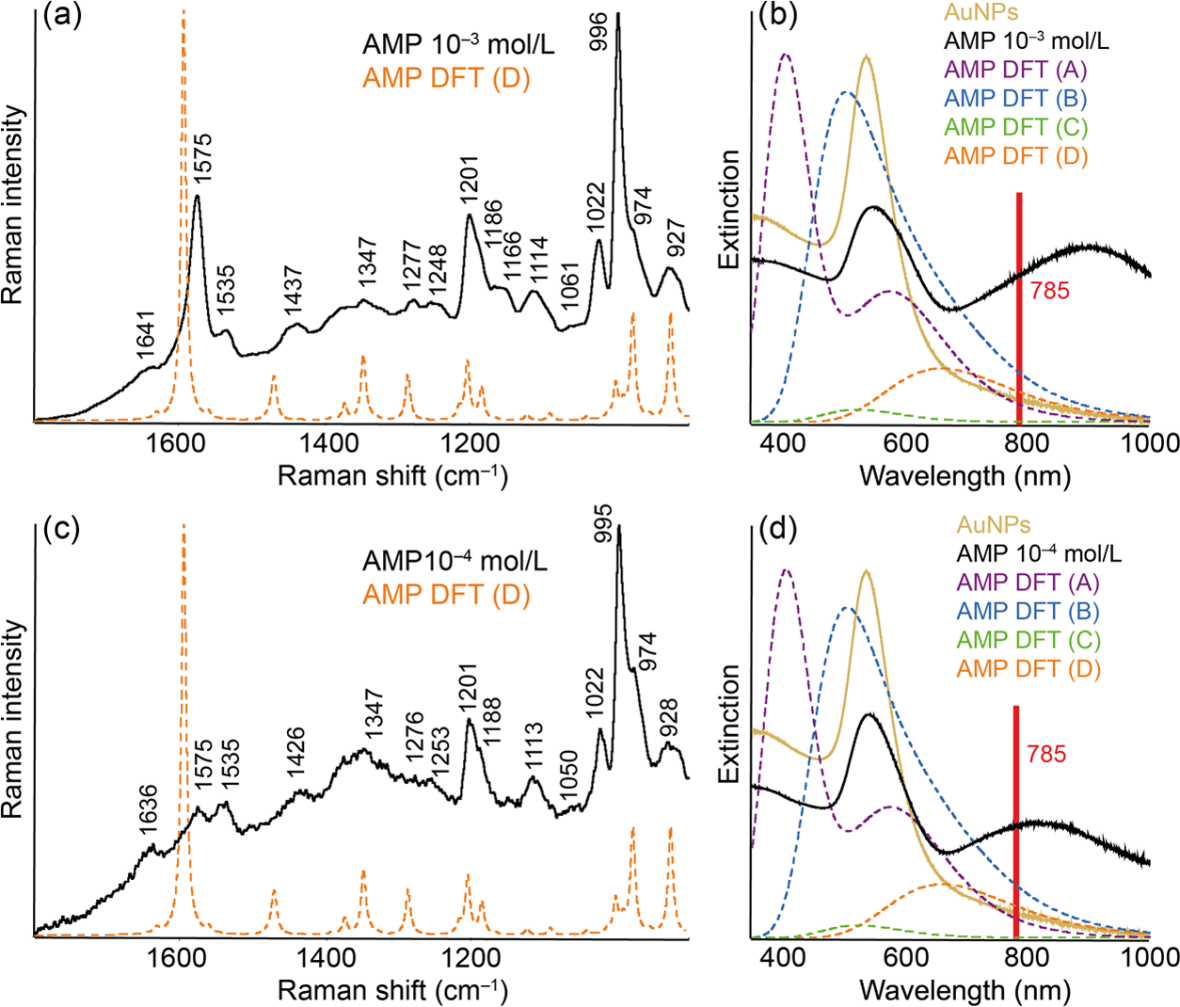
Experimental and calculated Au-SERS (a, c) and extinction (b, d) spectra of AuNPs modified by AMP of concentrations 10^–3^ mol/L (a, b) and 10^–4^ mol/L (c, d). The structures of the discussed molecular complexes A–D are included in the SI.

Au-SERS spectra of amphetamine are shown in Figure 2. In the case of the combination of AuNPs and AMP, it was possible to measure the SERS spectra at analyte concentrations of 10^–3^ and 10^–4^ mol/L. The signal of the molecules was well observable for both concentrations. In the case of the next lower tested concentration (10^–5^ mol/L), it was no longer possible to observe any signal arising from the molecules. The reason for these observations may be the change observed in the extinction spectra. The pure Au colloid shows a plasmon resonance maximum at 536 nm, which is in good agreement with the most frequently published cases of AuNP extinction spectra measurements.^33^ The addition of the two AMP concentrations mentioned above is manifested by a slight red shift of the maximum of this band. However, what is much more significant is that the addition of the analyte causes the appearance of a new band in the extinction spectra, the maximum of which is located at 819 nm for a lower concentration and 897 nm for a higher one.

In order to assess the possibility of the formation of “metal–molecule” surface complexes, DFT calculations of SERS and extinction spectra of these complexes were performed, taking into account several options of molecular interactions with the metal atom (Figure S1). These results are shown schematically in the right part of the figure. In the case of AMP, as well as other analytes, the possibility of forming a covalent bond of one Au atom to an amino group, a covalent bond of two Au atoms to an amino group, a noncovalent interaction of Au with an amino group, and a noncovalent interaction of Au with an aromatic ring was considered (in the figure, these variants are designated as A, B, C, and D, respectively). The given order of the complexes also corresponds to the increasing value of the maximum wavelengths of the respective complexes.

The figure shows changes in the extinction spectra before and after AMP modification. Based on the calculation, it can be stated that replacement of one of the covalently bonded hydrogen atoms in the amino group by a gold atom probably does not occur. Such a complex should manifest itself as a well-observable band located below the plasmon resonance maximum (405 nm). The plasmon resonance band itself expands noticeably compared to the unmodified colloid, and its intensity is indisputably lower. It cannot be excluded that both hydrogens of the amino group are replaced by gold atoms since the complex calculated in this way should show a maximum in the near region of the plasmon resonance (505 nm). In the same way, the possibility of noncovalent interaction of Au with the amino group cannot be completely debarred. Even the maximum of this complex would be located near the plasmon resonance maximum (524 nm). The highest ranked of the considered complexes is the one in which the noncovalent interaction of Au with the aromatic ring was considered. This complex, whose extinction maximum value corresponds to 660 nm, is therefore also the closest to the value of the used excitation wavelength. In addition, in the experimental extinction spectra of the modified colloid, we can observe the previously mentioned newly formed spectral maximum, which may solidly correspond to the last-mentioned complex. Although its real position is indisputably higher, this fact can be largely caused by the theoretical limitations of the calculation. It is also highly probable that in real systems, there is no interaction with only one Au atom, but with entire adatomic Au clusters, for which a more intense red shift is definitely predictable.^33^

The fact that the spectral profile of the simulated SERS spectrum of complex D best resembles the profile of the real SERS spectrum corresponds to the stated findings. The most intense bands in the experimental Au-SERS spectrum of AMP are the 1575, 1535, 1201, 1186, 1022, 996, 974, and 927 cm^–1^ bands. The complete assignment of the bands in the imaged region is shown in Table 1.

**Table 1:** Assignment of the Vibrational Modes of Au-SERS Spectra of AMP

Raman shift (cm^–1^)		
SERS (10^–3^ mol/L)	DFT (D)^a^	assignment of the vibrational modes
1641	1631	δ_sci_ (–NH_2_)
1575	1594	ν (C=C)_ar_
1535	1560	ν (C–C)_ar_
1437	1469	δ_sci_ (–CH_2_), δ (–CH)_ar_
1398	1390	δ_umb_ (–CH_3_)
1378	1372	δ (–CH), δ_twi_ (–CH_2_), δ_roc_ (–NH_2_)
1347	1347	δ (–CH), δ_wag_ (–CH_2_)
1277	1285	δ (–CH), δ_wag_ (–CH_2_)
1248	1214	δ_twi_ (–NH_2_), δ (–CH_3_), δ (–CH)
1201	1203	δ (–CH_2_), δ (–CH)_ar_, ν (C–C)_aryl_
1186	1184	δ_ip_ (–CH)_ar_
1166	1165	δ_ip_ (–CH)_ar_
1114	1122	δ_twi_ (–CH_2_), δ (–CH), δ (–CH_3_), δ (–NH_2_)
1093	1090	skeletal
1061	1039	skeletal
1022	1000	δ_oop_ (–CH)_ar_
996	990	δ (–CH)_ar_
974	976	δ (–CH_3_), δ (–NH_2_)
927	924	δ_oop_ (–CH)_ar_, δ (–CH_3_)

a The listed frequencies are scaled by the scaling factor *k* = 0.97, ν—stretching, δ—deformation, umb—umbrella, sci—scissoring, twi—twisting, wag—wagging, roc—rocking vibration, ar—aromatic, ip—in-plane, and oop—out-of-plane.

It follows from the table that, except for the 974 cm^–1^ band, all prominent bands belong to vibrational motions that are at least partially related to the aromatic ring. The most intense group of bands is at 1022, 996, and 927 cm^–1^, which all belong to out-of-plane vibrations. Assuming that AMP is attached to AuNPs primarily by the aromatic ring, this information is consistent with commonly considered surface selection rules^34^ since the direction of these vibrations is perpendicular to the nanoparticle surface. In accordance with the high intensity is also the fact that the aromatic ring is located closest to the surface; therefore, its vibrations are amplified the most.

The presence of bands belonging to the vibrations of the amino group in the spectra also confirms the assumption that in the case of AuNPs and AMP, there is probably no interaction through the amino group. However, it is not possible to definitely reject the hypothesis that the binding of the molecule occurs in a way different than the one indicated. However, due to the proximity of the absorption maximum of the resulting complex to the excitation wavelength, it is likely that the molecules bound in this way are the most amplified, while those bound otherwise become almost “invisible” or manifest themselves as weaker signals in the experimental spectra. The stated findings are the same for both investigated AMP concentrations; therefore, it is expected that at least in the studied concentration interval, AMP interacts with AuNPs in a similar way. The band assignments of the other considered complexes are part of the SI as Tables S3–S6.

**Figure 3:**
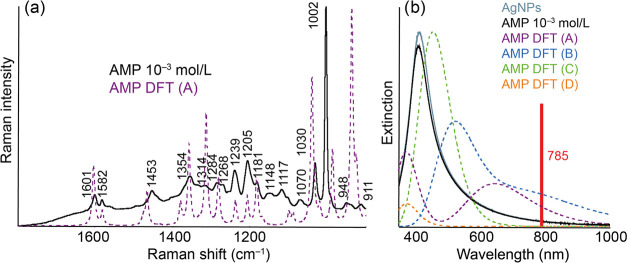
Experimental and calculated Ag-SERS (a) and extinction (b) spectra of AgNPs modified by AMP of concentration 10^–3^ mol/L. The structures of the discussed molecular complexes A–D are included in the SI.

Ag-SERS spectra are shown in Figure 3. In the case of AgNPs, it was possible to measure reliably only the higher of the tested concentrations, i.e., 10^–3^ mol/L. This is noteworthy because silver substrates generally exhibit a higher level of enhancement than gold.^5,6^ We can again search for an explanation in the extinction spectra of the system before and after AMP modification. The figure clearly shows that opposite AuNPs, where the modification of the AMP system was associated with a clearly observable change in the profile of the extinction spectrum, in the case of AgNPs, neither the course of the spectra nor their intensity differ much. As in the case of AMP-modified AuNPs, the same DFT calculations of SERS and extinction spectra were performed in the case of Ag, while the labeling of the considered complexes is the same as in the case of Au. The maxima of the calculated extinction spectra are 373 and 646 nm for one Ag atom covalently bound to an amine, 525 nm for two Ag atoms covalently bound to an amine, 456 nm for a noncovalently bound Ag atom to an amine, and 374 nm for noncovalently bound Ag to an aromatic ring. The results of the DFT calculations and the assignment table of the individual AMP bands for Ag are included in the SI as Figure S2 and Tables S7–S10. From the obtained results, it could be stated that in the case of AgNPs and AMP, we suggest that the covalent interaction through the amino group is most probably the observed binding option. The computed spectral profile of the A complex is most like the experimental data, although the agreement level is not so high, as in the case of Au-SERS of AMP. That could be because a small number of molecules actually form the “A-type” complexes, making the resulting signal much more “averaged” over all types of randomly arranged molecules.

### SERS of Methamphetamine

**Figure 4:**
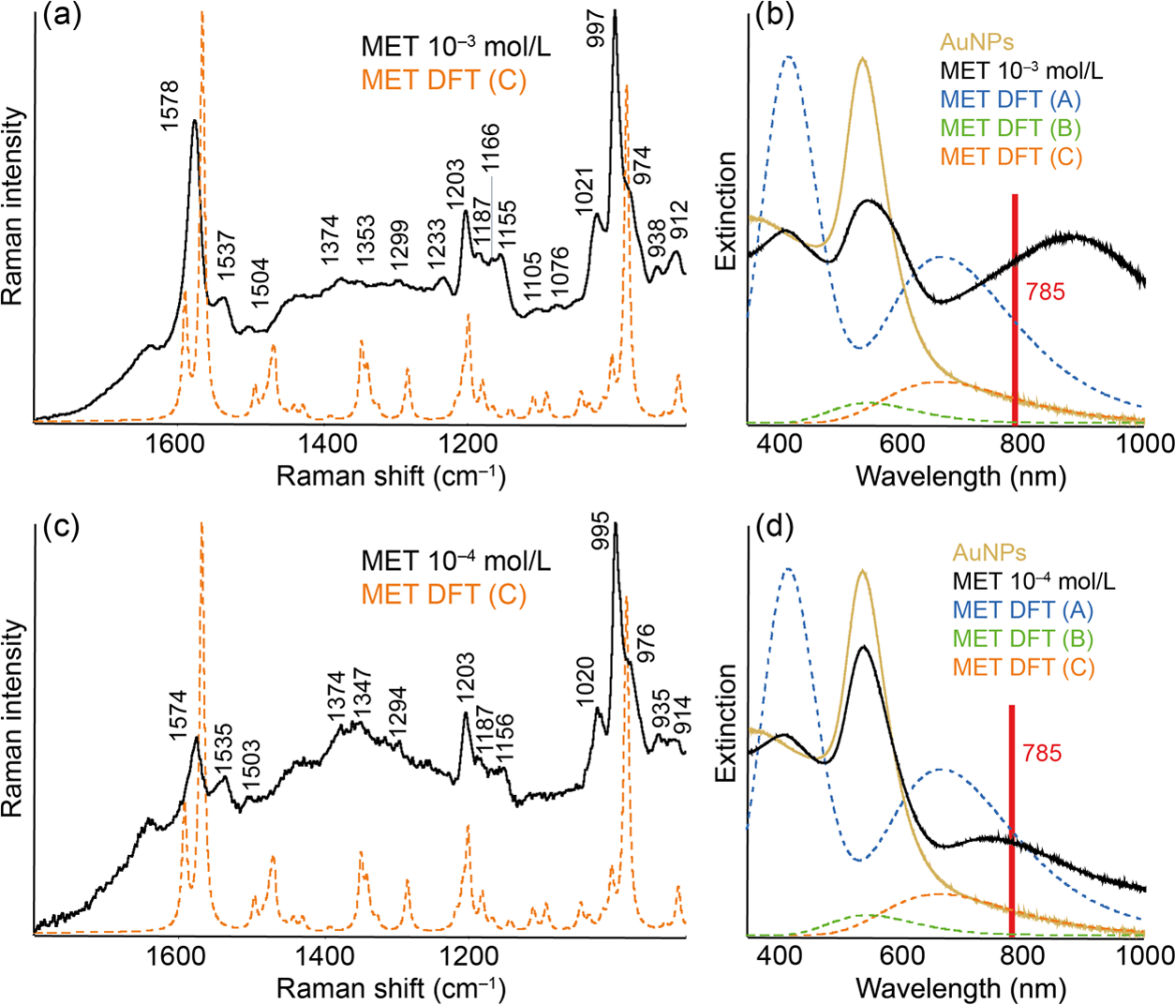
Experimental and calculated Au-SERS (a, c) and extinction (b, d) spectra of AuNPs modified by MET of concentrations 10^–3^ mol/L (a, b) and 10^–4^ mol/L (c, d). The structures of the discussed molecular complexes A–C are included in the SI.

Au-SERS spectra of methamphetamine are shown in Figure 4. As in the case of AMP, it was possible to measure SERS spectra for MET-modified AuNP systems with concentrations of 10^–3^ and 10^–4^ mol/L. In this case, the observed trend corresponds to the observations made in the extinction spectra. During the modification of AuNPs, two new maxima appear in the extinction spectra, for both investigated concentrations of MET. These maxima are located at 405 and 871 nm for the higher investigated concentration and at 405 and 741 nm for the lower concentration. Likewise, the main band in the extinction spectra shifts to higher wavelength values, namely, 545 and 536 nm for a concentration of 10^–3^ and 10^–4^ mol/L, respectively.

The maximum values of the simulated extinction spectra of the complexes are 418 and 669 nm for complex A (covalent interaction of the amino group and Au), 548 nm for complex B (noncovalent interaction of the amino group and Au), and 666 nm for complex C (noncovalent interaction of the aromatic ring with Au) (Figure S3). When compared with the experimentally obtained spectra, it can be concluded that the formation of all three considered complexes is possible. The position of the lower of the newly formed bands (405 nm) corresponds well to the lower band of complex A, and the position of the higher of them approximately corresponds to the maximum of the second band of this complex. The formation of complex B could be the cause of the shift of the maximum and the broadening of the original plasmon resonance band, which is very well observable. Likewise, the position of the band of complex C within the uncertainty of the calculation corresponds to the position of the higher of the newly formed bands (871 nm).

However, when comparing the experimental and DFT Au-SERS spectra, the most similar to the experiment was the DFT spectrum of complex C, i.e., the noncovalent interaction of the aromatic ring with the Au atom. It is a question of why a larger form is not observed with the spectrum belonging to complex A, which has an extinction maximum in a similar region and whose formation probably occurs, which can be stated based on the presence of a band at 405 nm in the extinction spectra. It is possible that this covalent interaction causes an inappropriate orientation of the rest of the molecules toward the Au surface, which is demonstrated by weakened bands of the in-plane modes. Thus, these complexes would become more or less invisible, and the spectral profile would be largely determined by the profile of the C complexes as observed in the experimental data.

In the experimental Au-SERS spectrum, there are dominant bands at 1578 and 1537 cm^–1^ and groups of bands around 1200 and 1000 cm^–1^. As in the case of AMP, all of the named bands include an aromatic ring in their movements. The most intense is the band at 997 cm^–1^, which belongs to the out-of-plane deformation vibration of the ring and which is thus in an ideal position as far as the validity of the surface selection rules is concerned. The complete assignment of MET bands in the studied spectral interval is shown in Table 2, and the band assignments of the other considered complexes are part of the SI as Tables S11–S13.

**Table 2:** Assignment of the Vibrational Modes of Au-SERS Spectra of MET

Raman shift (cm^–1^)		
SERS (10^–3^ mol/L)	DFT (C)^a^	assignment of the vibrational modes
1578	1591	ν (C–C)_ar_
1537	1567	ν (C=C)_ar_
1504	1494	δ_sci_ (–CH_3_)_N_
1374	1347	δ_wag_ (–CH_2_), δ (–CH)
1353	1339	δ_twi_ (–CH_2_), δ (–CH), δ (–CH)_ar_, δ (–NH)
1299	1283	δ_wag_ (–CH_2_), δ (–CH)
1233	1216	δ_twi_ (–CH_2_), δ (–CH)_ar_, δ (–CH_3_)_C_
1203	1207, 1199	δ (–CH_3_)_C,N_, δ_twi_ (–CH_2_), δ (–CH), δ (–CH)_ar_, ν (C–C), ν (C–N),
1187	1180	δ_ip_ (–CH)_ar_
1166	1165	δ_ip_ (–CH)_ar_
1155	1141	ν (C–N), δ_twi_ (–CH_2_), δ (–CH), δ (–CH_3_)_N_
1105	1109	δ_twi_ (–CH_2_), δ (–CH), δ (–CH_3_)_N_
1076	1091	skeletal vibration
1021	1013	δ (–CH)_ar_, ν (C–C)_ar_, ν (C=C)_ar_
997	1001	δ_oop_ (–CH)_ar_
974	980	δ_oop_ (–CH)_ar_
938	927	δ_oop_ (–CH)_ar_, δ (–CH_3_)_C_
912	909	δ (–CH_3_)_C_, ν (C–N)

a The listed frequencies are scaled by the scaling factor *k* = 0.97, ν—stretching, δ—deformation, sci—scissoring, twi—twisting, wag—wagging, roc—rocking vibration, ar—aromatic, ip—in-plane, and oop—out-of-plane.

**Figure 5:**
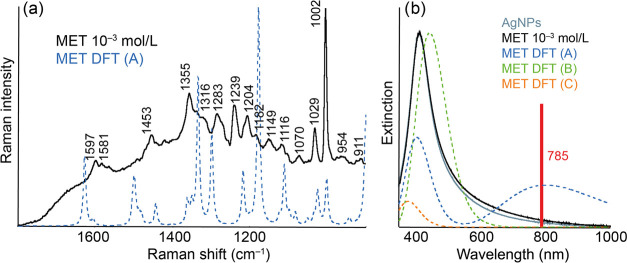
Experimental and calculated Ag-SERS (a) and extinction (b) spectra of AgNPs modified by MET of concentration 10^–3^ mol/L. The structures of the discussed molecular complexes A–C are included in the SI.

Ag-SERS spectra of MET are plotted in Figure 5. Probably due to the considerable structural similarity with AMP, even in the case of Ag-SERS MET, only the higher of the investigated concentrations proved to be active.

In the calculation of the DFT extinction spectra, identical complexes were considered, as in the case of Au. In the case of complex A, the calculated spectrum shows maxima at 405 and 803 nm for complex B at 443 nm and for complex C at 377 nm. As in the case of AMP, the addition of MET to AgNPs does not appear to fundamentally affect the character of the extinction spectrum, as can be seen in the figure. Due to the position of the excitation wavelength (785 nm), the possibility of excitation of the emerging complex A (if it actually occurs) appears to be the most likely. This fact correlates with the fact that even with respect to the calculated SERS spectra, the spectrum of complex A is the most similar to the experimentally obtained spectra, although this agreement is again noticeably smaller compared to that of the Au system. It is possible that due to the smaller number of emerging complexes than in the case of Au (small changes in the extinction spectra of the Ag system), most of the molecules are oriented randomly or even in layers. Even a relatively exact match of the position of the extrinsic radiation with the calculated maximum of complex A is not sufficient to completely “rewrite” the profile of the resulting spectrum, where the existence of groups and differently oriented molecules is much more evident. The results of the DFT calculations and the assignment table of the individual MET bands for Ag are included in the SI as Figure S4 and Tables S14–S16.

As in the case of Au systems, and also in the case of Ag, the most dominant bands in the experimental SERS spectrum are at 1029 and 1002 cm^–1^, similar to the case of AMP-modified AgNPs. This fact needs to be kept in mind for possible analytical use because the SERS spectra of AMP and MET presented in this work are very similar due to their very close chemical structure.

### SERS of Methylenedioxymethamphetamine

**Figure 6:**
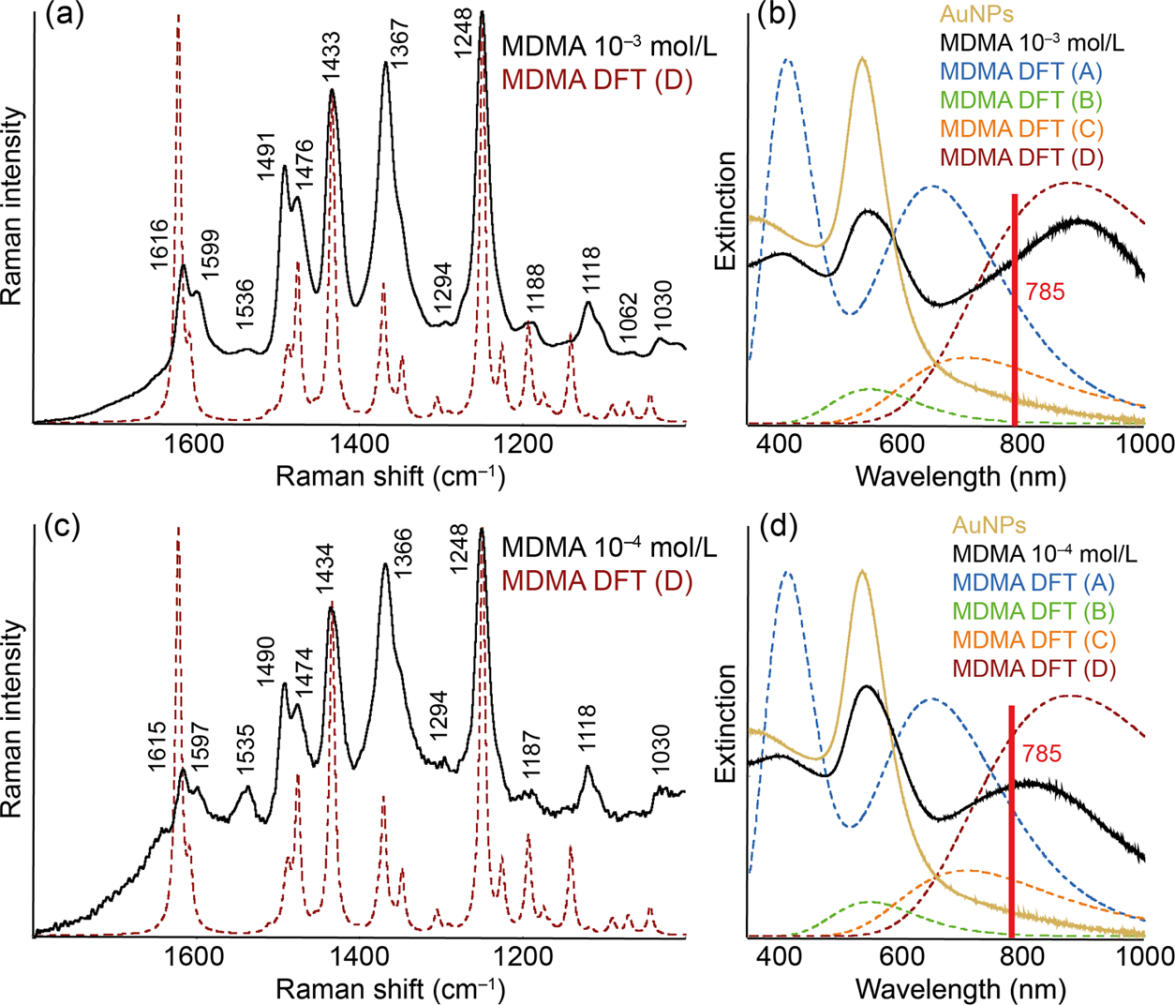
Experimental and calculated Au-SERS (a, c) and extinction (b, d) spectra of AuNPs modified by MDMA of concentrations 10^–3^ mol/L (a, b) and 10^–4^ mol/L (c, d). The structures of the discussed molecular complexes A–D are included in the SI.

Au-SERS spectra of methylenedioxymethamphetamine (MDMA) are shown in Figure 6. Even in the case of MDMA, it was possible to measure the mentioned concentrations. Since MDMA’s structure is significantly different from AMP and MET, it was necessary to take this fact into account when considering the resulting complexes. In the case of MDMA, complex A belongs to the covalent interaction of Au with the amino group, complex B to the noncovalent interaction of the amino group to Au, complex C to the noncovalent interaction with the aromatic ring, and complex D to the noncovalent interaction with the dioxin part (Figure S5).

For the mentioned calculated complexes, the maxima of the absorption spectra are characteristic at 412 and 650 nm (complex A), 547 nm (complex B), 707 nm (complex C), and 877 nm (complex D). Even in the case of modification of AuNPs with MDMA, the profile of their extinction spectrum changes. New maxima appear for MDMA concentrations of 10^–3^ mol/L (404 and 890 nm) and 10^–4^ mol/L (395 and 819 nm), as well as a shift of the maximum of the already present band (541 nm for higher and 542 nm for lower concentrations of MDMA). Also, in the case of MDMA, the possibility of covalent interaction should be taken into account because of the 400 nm band’s appearance in the modified colloid extinction spectra. Considering the wavelength of the laser, molecules bound to the Au surface through the oxygenated part of the structure should be the most visible in the resulting spectra, based on experimental and calculated data.

This assumption is in very good agreement with the experimental SERS data. The calculated SERS spectrum of complex D corresponds to the experimentally obtained spectra, while this spectrum is dominated by bands at 1490, 1474, 1434, 1366, and 1248 cm^–1^. All of these vibrational modes include the vibrations of the –CH_2_ group close to the oxygen atoms. We believe that this fact confirms the assumption of interaction through this structural part because considered vibrations are then closest to the surface, and in most cases, their movement is kept in a direction perpendicular to the Au surface. The detailed assignment of individual bands in the monitored spectral interval is shown in Table 3, and the band assignments of the other complexes considered are part of the SI as Tables S17–S20.

**Table 3:** Assignment of the Vibrational Modes of Au-SERS Spectra of MDMA

Raman shift (cm^–1^)		
SERS (10^–3^ mol/L)	DFT (D)^a^	assignment of the vibrational modes
1616	1622	ν (C–C)_ar_
1599	1608	ν (C=C)_ar_
1490	1487	δ_sci_ (–CH_2_)_O_, δ_sci_ (–CH_2_)_ar_, δ_ip_ (–CH)_ar_
1474	1475	δ_sci_ (–CH_2_)_O_, δ_ip_ (–CH)ar
1434	1433	δ_sci_ (–CH_2_)_O_, δ_ip_ (–CH)_ar_, δ_twi_ (–CH_2_)_ar_, ν (C=C)_ar_
1366	1370	δ_wag_ (–CH_2_)_O_, δ_ip_ (–CH)_ar_, δ_twi_ (–CH_2_)_ar_, ν(C–C)_ar_
1347	1347	δ (–CH_2_)_ar_, δ (–CH)
1248	1249	ν (C–O), ν (C–C)_ar_, ν (C=C)_ar_, δ (–CH_2_)_ar_
1192	1193	δ_twi_ (–CH_2_)_O_, δ_ip_ (–CH)_ar_
1140	1117	δ_ip_ (–CH)_ar_
1070	1070	skeletal vibration
1042	1043	δ (–CH_3_)_N_, δ (–CH_3_)_C_, δ (–NH)

a The listed frequencies are scaled by the scaling factor *k* = 0.98, ν—stretching, δ—deformation, sci—scissoring, twi—twisting, wag—wagging, roc—rocking vibration, ar—aromatic, ip—in-plane, and oop—out-of-plane.

**Figure 7:**
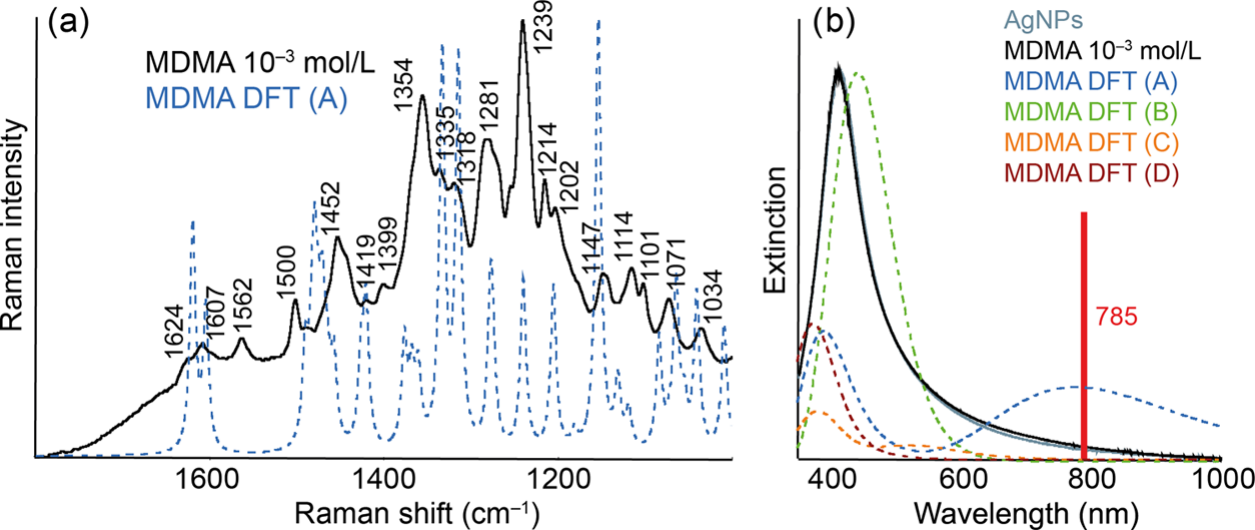
Experimental and calculated Ag-SERS (a) and extinction (b) spectra of AgNPs modified by MDMA of concentration 10^–3^ mol/L. The structures of the discussed molecular complexes A–D are included in the SI.

Ag-SERS spectra of MDMA are shown in Figure 7. Even in the case of MDMA, it was not possible to measure a lower concentration than 10^–3^ mol/L. This result can be attributed to the fact that after the modification of AgNPs, there were no noticeable changes in the extinction spectrum and therefore probably not even the formation of a larger number of complexes. The maxima of the absorption bands for the calculated complexes with Ag are 391 and 779 nm (complex A), 443 nm (complex B), 381 nm (complex C), and 374 nm (complex D). The manifestation of those molecules that are covalently bound through the amino group appears to be the most likely. This corresponds to the highest agreement between the calculated SERS spectrum of this complex and the experimental SERS spectra, although, as in the cases of AMP and MET, there are non-negligible differences in this comparison, and the reason for this will probably be the same as the reasons mentioned for the previous two molecules. The results of the DFT calculations and the assignment table of the individual MDMA bands for Ag are included in the SI as Figure S6 and Tables S21–S24.

### Narrow Interval Chemical Enhancement

The results from the previous sections show that, especially in the case of used gold colloids, the addition of analytes causes significant changes in the profile of the measured extinction spectra of AuNPs. In the case of AgNPs, these changes were barely observable; therefore, we focused on AuNPs in our further investigation. As it was said earlier, we believe the formation of various “molecule–metal” surface complexes to be the reason for the changes in the profiles of the extinction spectra, as a similar correlation was already published before.^35^ We observed that the intensity, position, and shape of the newly formed bands depend on the concentration of the added substance. While in the previous part of the text, we dealt with the connection between the measured extinction spectra and the overall appearance of the SERS spectra, in this section, we will focus on the subtle differences in the SERS spectra profiles changes caused by the appearance of the new extinction maxima in the drug-modified systems.

In selected spectral intervals, we chose several bands whose areas we compared with each other. We assume that while maintaining the same method of binding of the analyte to the surface of the nanoparticles, the ratio of the individual bands’ areas should be preserved when the concentration changes, i.e., the relative intensity of the bands in the SERS spectrum is concentration-independent. However, even a cursory survey of the profiles of the experimental SERS spectra makes it clear that this is not the case. We believe that this situation is largely caused by the fact that the newly emerging bands in the extinction spectra are located close to the position of the excitation wavelength (785 nm). In further considerations, we will start from the assumption that the resulting SERS intensity is given by the well-known eq 1, where inc refers to incidental and s refers to Stokes-scattered radiation.^5^
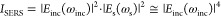
1

The given approximation, where the intensity of the scattered field is equal to the intensity of the incidental field, is commonly considered when one is studying SERS in the higher concentration, assuming that the minor deviations caused by neglecting the difference between the field’s intensities would manifest themselves more significantly in the special cases, such as single molecule experiments. We would like to demonstrate that even in the case of higher-concentration SERS, these effects may occur, leading to observable changes in the area’s ratio of the SERS bands.

**Figure 8:**
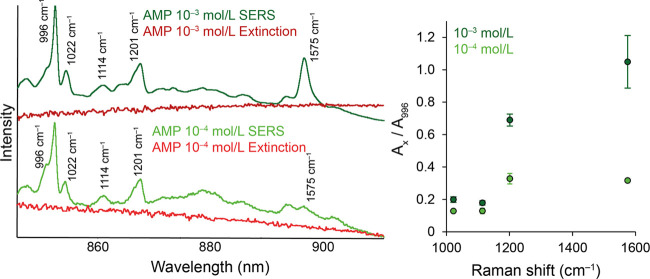
Comparison of concentration-dependent spectral profiles of Au-SERS spectra and extinction spectra of AMP-modified AuNPs and the trend of area ratios of selected SERS bands.

Our evaluation approach for the AMP-modified systems is presented in Figure 8. In the left part of the figure, we show the SERS and extinction spectra of the systems at different concentrations in the respective wavelength regions. The value of the Raman shift is also given for the bands discussed further for a better orientation. In the right part of the figure, the trend of the areas’ ratio of labeled bands to the 996 cm^–1^ band’s area is shown. Error bars have the value of the sample standard deviation obtained from five measurements in both the positive and negative directions.

The fact that the relative area of the band at 1575 cm^–1^ varies considerably depending on the concentration of AMP used can be observed with the naked eye. It can also be seen that the profile of the extinction spectra of the respective systems differs in this region. While in the case of the concentration 10^–3^ mol/L, a newly formed extinction band is located in the region of the 1575 cm^–1^ band almost at its maximum, in the case of the concentration 10^–4^ mol/L, the maximum of the discussed band is found at lower wavelengths. The values of the area ratios of the considered bands (1022, 1114, 1201, and 1575 cm^–1^) to the 996 cm^–1^ band for both measured concentrations are shown in the graph in Figure 8. The observed trend reveals that as the wavelength of the inelastically scattered photons increases, the relative intensity of the corresponding surface vibrational modes changes. The biggest difference is thus observed in the case of the already mentioned band at 1575 cm^–1^.

Since the increase in relative intensity occurs only in a relatively narrow interval of wavelengths and is probably caused by the formation of “molecule–metal” complexes, we will refer to this phenomenon as narrow interval chemical enhancement (NICE). When attempting a semiquantitative evaluation of this phenomenon, we started from the assumption that when relating the area of a specific band to the 996 cm^–1^ band’s area (for the case of AMP), at the same time, we normalized the areas of these bands, as a result of which we should eliminate the concentration dependence of the intensity. Of course, it considers the fact that the resulting SERS intensity was governed by eq 1. It is definitely dependent on the intensity of the incident radiation and on the frequency of this radiation, especially with respect to the possible resonance with transitions in molecular complexes. We believe, however, that such resonances with excitation radiation would contribute to the amplification of the intensity of the bands of all vibrations equally and therefore this variability is also suppressed by our normalization. Considering the validity of the above-mentioned assumptions, we believe that the changes discussed below are mainly caused by a different degree of inelastically scattered photon resonance with transitions in molecular complexes. We then calculate this particular gain using eq 2.
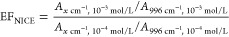
2

Applying the EF_NICE_ calculation to the AMP case, we obtain, as expected, a value that increases with the wavelength of the scattered radiation. In the case of the 1575 cm^–1^ band, it reaches a value higher than 3 (the dependence of the EF_NICE_ values on the studied AMP band shifts can be found in Figure S7). It is also noteworthy that in the highest-placed band case, the value of the error bars is significantly larger. We believe that this is caused by the different rates of occurrence of the considered complexes in the path of the laser beam during a specific measurement.

**Figure 9:**
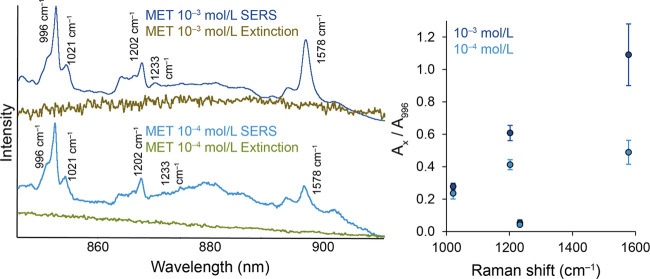
Comparison of concentration-dependent spectral profiles of Au-SERS spectra and extinction spectra of MET-modified AuNPs and the trend of area ratios of selected SERS bands.

The situation for the modification of AuNPs by MET is presented in Figure 9. Even in the case of this molecule, the change in relative intensities is most noticeable at the highest investigated band, i.e., 1578 cm^–1^. The courses of the extinction spectra are highly correlated with those from the AMP-modified systems. For the higher of the investigated concentrations, the wavelengths of the inelastically scattered photons correspond to the maximum of the discussed band of the extinction spectrum. For the lower of the concentrations, these wavelengths are above the value of the extinction maximum. The development trend of band ratio values also correlates with the AMP case. As the wavelength increases, the difference between the values of the band ratios for individual concentrations increases, as does the sampling standard deviation from these measurements. The highest EF_NICE_ value is reached again for the band at 1578 cm^–1^, and in the case of MET, this value is higher than 2. The dependence of the EF_NICE_ values on the studied MET band shifts can be found in Figure S8.

**Figure 10:**
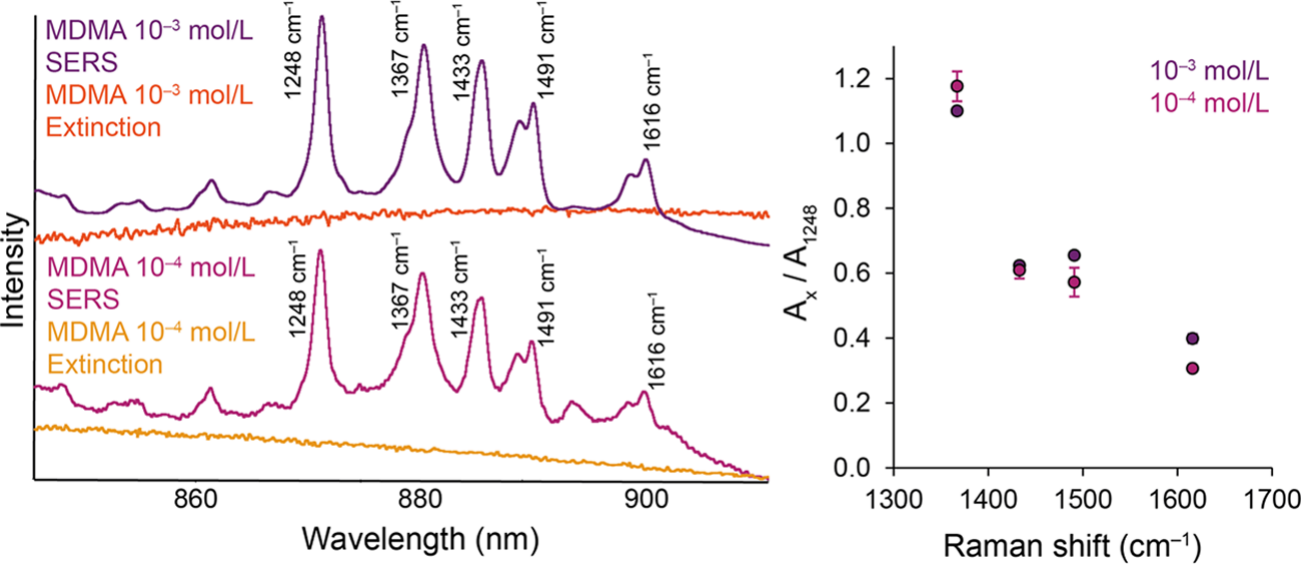
Comparison of concentration-dependent spectral profiles of Au-SERS spectra and extinction spectra of MDMA-modified AuNPs and the trend of area ratios of selected SERS bands.

Finally, the selected spectral regions for the MDMA-modified AuNP systems are shown in Figure 10. As in the previous cases, there is also an apparent increase in the relative intensity of the highest-ranked MDMA band, although this increase is the least noticeable of the three analyzed molecules. The growth of the relative intensities of the SERS bands again is consistent with the trend in the extinction spectra of the individual systems. The trend of the differences between the concentration-dependent ratios of the band’s areas also corresponds to the previous findings, although these changes are not so significant in terms of magnitude. The value of the EF_NICE_ factor for the highest investigated band is approximately 1.3 in the case of MDMA. The dependence of the EF_NICE_ values on the studied MDMA band shifts can be found in Figure S9.

### Excitation Wavelength Dependence

Finally, the excitation wavelength dependence in the relevant near-infrared region was studied. For this purpose, an excitation wavelength of 1064 nm was used for the acquisition of all investigated amphetamines’ SERS spectra. The mentioned comparison for Au-SERS of AMP is displayed in Figure S10. At the first glance, it is obvious that while in the case of the higher used analyte concentration (10^–3^ mol/L), the Au-SERS spectra are quite similar for both excitation wavelengths, for the lower concentration (10^–4^ mol/L), they significantly differ. Particularly, the area ratio of bands around 1600 cm^–1^ underwent significant changes, and there is also a noticeable increase of a 1426 cm^–1^ band relative intensity. Most significant changes were observed for the bands at 1201, 1022, and 996 cm^–1^, whose intensity noticeably diminished. On the contrary, a new vibrational band at 1032 cm^–1^ arises. We believe that this concentration-dependent difference is caused by the related extinction spectra’s profile. Although it is not possible to measure its profile so far into the near-infrared region, it can be hypothesized (based on the spectral course up to 1000 nm) that for the lower AMP concentration, the intensity of extinction will decrease much faster toward the 1064 nm excitation wavelength than for the higher one. Therefore, we assume that for the lower one AMP concentration, most of the radiation (both excitation and scattered) is located out of the modified nanoparticles’ newly formed extinction band, whereas for the higher one, the location of the related extinction band is much more favorable, thus resulting in the appearance of a much more similar one to the one recorded when using a lower excitation wavelength. However, after thorough scrutinization, a difference in the bands’ area ratios for two used excitation wavelengths could be found (Figure S11). Generally, the ratio of investigated bands is lower for the excitation wavelength of 1064 nm, while this effect is most noticeable for the highest concerned band (1575 cm^–1^). This observation could support the hypothesis about high enhancement caused by additional resonance, which has been put forth in the previous section.

As for the MET-modified systems (Figure S12), a comparison of excitation wavelengths brings results similar to those for the AMP-modified NPs. Even in this case, spectral profiles are more similar for the higher used concentration, while for the lower one, the spectral course significantly differs. This corresponds to the course observed in the extinction spectra, which is very similar to the AMP-modified ones. Even for MET, differences between the bands’ area ratios when using different excitation wavelengths were recorded (Figure S13). As before, the largest difference was observed for the band at 1578 cm^–1^.

MDMA Au-SERS spectra measured with excitation wavelengths of 785 and 1064 nm are displayed in Figure S14. The differences between applied analyte concentrations are not so striking as for AMP and MET, although even in this case several changes can be observed, for example, the overall difference in the bands’ area ratio. There is also a high increase of the 1030 cm^–1^ band’s relative intensity, while bands located higher than 1430 cm^–1^ seem to be gradually diminishing. Also, the band at 1118 cm^–1^ practically disappeared. We assume that all of these changes could be again attributed to the courses in extinction spectra. It should be noted that for both MDMA concentrations, extinction bands of our interests have their maximum slightly higher than AMP and MET, while for the 10^–4^ mol/L concentration of MDMA, the related band is also more intense than in the spectra of other two analytes. This correlates with the profile of Au-SERS spectra recorded when using this concentration, as they clearly have a higher value of the signal-to-noise ratio, which we attribute to the fact that the related extinction band reaches far into the area of the near-infrared region. Also, a change in the bands’ area ratios when using different excitation wavelengths and an analyte concentration of 10^–3^ mol/L was observed (Figure S15).

As for the Ag-SERS spectra, not much amount of information could be extracted, even from the systems modified by the concentration of 10^–3^ mol/L. When measuring with the excitation wavelength of 1064 nm, the AMP signal generally was not observed, although the profile of the background proposes that it is somehow affected by the AMP’s presence (Figure S16). The situation is slightly better for the MET-modified systems, where bands at 1002, 1029, 1453, 1581, and 1597 cm^–1^ could be observed (Figure S17). In the Ag-SERS spectra of MDMA-modified systems, only bands at around 1034 cm^–1^ are present (Figure S18). Courses of all related extinction spectra imply that this will probably be caused by the incomparably lower effect of the analytes on the AgNPs, which even more emphasizes the necessity of such a phenomenon for the needs of the analysis of the mentioned substances.

## Conclusions

In conclusion, this study sheds light on the crucial role of chemical enhancement in the surface-enhanced Raman scattering (SERS) spectroscopy analysis of amphetamine–metal interactions. The findings demonstrate the potential of SERS in detecting amphetamine-based addictive stimulants, particularly in the concentration range from 10^–3^ to 10^–4^ mol/L. The formation of molecular complexes and the excitation wavelength significantly influence the resulting SERS spectra, with complexes exhibiting extinction bands in the region of the excitation wavelength dominating the observed spectral profile. It is important to emphasize that the way different molecules interact with a particular substrate varies. For example, it seems that in the case of the drug-modified AuNPs, the covalent interaction is more probable for MET and MDMA, and so for the analytes with secondary amines in its structure. However, this assumption needs to be confirmed by future comparative experiments.

Furthermore, a detailed assignment of vibrational modes to the observed SERS bands was achieved, particularly for molecules adsorbed on gold nanoparticles, with the best agreement obtained for the system modified with MDMA. This information provides valuable insights for future applications involving similar binding patterns or in the cases where molecules bind in a comparable manner.

Notably, the selective amplification of specific bands within a narrow interval of the inelastically scattered photon wavelengths is observed, influenced by the individual system extinction spectra profiles, whereby the importance of a suitably chosen spectral interval is demonstrated by comparing the two excitation wavelengths. While this phenomenon has implications in analytical chemistry, it is important to consider the potential nonlinearities and errors that may arise in calibration series due to the several-fold increase in the intensity of these bands. The findings presented in this study contribute to the fields of spectroscopy, physical chemistry, and drug analysis, offering valuable insights for forensic applications and a deeper understanding of the special case of chemical enhancement phenomena. It is also noteworthy that in our case, the AuNPs exhibit a higher SERS activity than AgNPs, which further emphasizes the importance of the chemical mechanism effect even for the analytical purpose. The knowledge gained from this research paves the way for further advancements in the detection and analysis of addictive substances, ultimately aiding law enforcement and forensic laboratories in their efforts to combat the challenges posed by the illicit drug market.

## Supporting Information Available

Nanoparticles preparation, molecular structures (DFT); calculated SERS and absorption spectra; vibrational assignments of other possible complexes; and SERS spectra measured with an excitation wavelength of 1064 nm (PDF)
