# Supplementary material for: Unveiling the Crucial Role of Chemical Enhancement in the SERS Analysis of Amphetamine–Metal Interactions on Gold and Silver Surfaces: Importance of Selective Amplification of the Narrow Interval of Vibrational Modes
Source: Anal Chem. 2024 Mar 7;96(14):5416–27. doi: 10.1021/acs.analchem.3c05189 (PMC11007674; doi:10.1021/acs.analchem.3c05189)
Supplement: Supplementary file 1 — ac3c05189_si_002.pdf [file ac3c05189_si_002.pdf]

Supporting Information

Unveiling the Crucial Role of Chemical Enhancement in SERS Analysis of Amphetamine-Metal Interactions on Gold and Silver Surfaces – Importance of Selective Amplification of the Narrow Interval of Vibrational Modes

Valerie Smeliková[a,b,† * ‡], Ivan Kopal[a,# ‡], Martin Člupek[b], Marcela Dendisová[a], Marie Švecová[b]

[a] Department of Physical Chemistry, University of Chemistry and Technology Prague, Technická 5, 166 28 Prague 6, Czech Republic

[b] Department of Analytical Chemistry, University of Chemistry and Technology Prague, Technická 5, 166 28 Prague 6, Czech Republic

[1] Both authors contributed equally

* Corresponding author: tel.: + 420 220 443 694, e-mail: smelikov@vscht.cz

Table of Content

Table S1 Reactants and their concentrations and volumes used for the gold colloids preparation and subsequent modification S4

Table S2 Reactants and their concentrations and volumes used for the silver colloids preparation and subsequent modification S5

Figure S1. Structure, DFT Raman and UV-Vis spectra of the considered Au-AMP complexes. S6

Table S3. Assignment of the vibrational modes of DFT spectra of Au–AMP complex A S7

Table S4. Assignment of the vibrational modes of DFT spectra of Au–AMP complex B S8

Table S5. Assignment of the vibrational modes of DFT spectra of Au–AMP complex C S9

Table S6. Assignment of the vibrational modes of DFT spectra of Au–AMP complex D S10

Figure S2. Structure, DFT Raman and UV-Vis spectra of the considered Ag-AMP complexes. S11

Table S7. Assignment of the vibrational modes of DFT spectra of Ag–AMP complex A S12

Table S8. Assignment of the vibrational modes of DFT spectra of Ag–AMP complex B S13

Table S9. Assignment of the vibrational modes of DFT spectra of Ag–AMP complex C S14

Table S10. Assignment of the vibrational modes of DFT spectra of Ag–AMP complex D S15

Figure S3. Structure, DFT Raman and UV-Vis spectra of the considered Au-MET complexes. S16

Table S11. Assignment of the vibrational modes of DFT spectra of Au–MET complex A S17

Table S12. Assignment of the vibrational modes of DFT spectra of Au–MET complex B S18

Table S13. Assignment of the vibrational modes of DFT spectra of Au–MET complex C S19

Figure S4. Structure, DFT Raman and UV-Vis spectra of the considered Ag-MET complexes. S20

Table S14. Assignment of the vibrational modes of DFT spectra of Ag–MET complex A S21

Table S15. Assignment of the vibrational modes of DFT spectra of Ag–MET complex B S22

Table S16. Assignment of the vibrational modes of DFT spectra of Ag–MET complex C S23

Figure S5. Structure, DFT Raman and UV-Vis spectra of the considered Au-MDMA complexes... S24

Table S17. Assignment of the vibrational modes of DFT spectra of Au–MDMA complex A S25

Table S18. Assignment of the vibrational modes of DFT spectra of Au–MDMA complex B S26

Table S19. Assignment of the vibrational modes of DFT spectra of Au–MDMA complex C S27

Table S20. Assignment of the vibrational modes of DFT spectra of Au–MDMA complex D S28

Figure S6. Structure, DFT Raman and UV-Vis spectra of the considered Ag-MDMA complexes... S29

Table S21. Assignment of the vibrational modes of DFT spectra of Ag–MDMA complex A S30

Table S22. Assignment of the vibrational modes of DFT spectra of Ag–MDMA complex B S31

Table S23. Assignment of the vibrational modes of DFT spectra of Ag–MDMA complex C S32

Table S24. Assignment of the vibrational modes of DFT spectra of Ag–MDMA complex D S33

Figure S7. NICE enhancement factor values for AMP-modified AuNPs. S34

Figure S8. NICE enhancement factor values for MET-modified AuNPs. S34

Figure S9. NICE enhancement factor values for MDMA-modified AuNPs. S35

Figure S10. Experimental Au-SERS (a,c) and extinction (b,d) spectra of AuNPs modified by MET of concentration 10^{-3} mol/L (a, b) and 10^{-4} mol/L (c, d). SERS spectra measured with different wavelengths does not share the same scale for better readability. S36

Figure S11. Comparison of excitation wavelength-dependent trends of SERS area ratios of selected AMP Au-SERS bands. S36

Figure S12. Experimental Au-SERS (a,c) and extinction (b,d) spectra of AuNPs modified by MET of concentration 10^{-3} mol/L (a, b) and 10^{-4} mol/L (c, d). SERS spectra measured with different wavelengths does not share the same scale for better readability. S37

Figure S13. Comparison of excitation wavelength-dependent trends of SERS area ratios of selected MET Au-SERS bands. S37

Figure S14. Experimental Au-SERS (a,c) and extinction (b,d) spectra of AuNPs modified by MDMA of concentration 10^{-3} mol/L (a, b) and 10^{-4} mol/L (c, d). SERS spectra measured with different wavelengths does not share the same scale for better readability. S38

Figure S15. Comparison of excitation wavelength-dependent trends of SERS area ratios of selected MDMA Au-SERS bands. S38

Figure S16 Experimental Ag-SERS (a) and extinction (b) spectra of AgNPs modified by AMP of concentration 10^{-3} mol/L. SERS spectra measured with different wavelengths does not share the same scale for better readability. S39

Figure S17. Experimental Ag-SERS (a) and extinction (b) spectra of AgNPs modified by MET of concentration 10^{-3} mol/L. SERS spectra measured with different wavelengths does not share the same scale for better readability. S39

Figure S18. Experimental Ag-SERS (a) and extinction (b) spectra of AgNPs modified by MDMA of concentration 10^{-3} mol/L. SERS spectra measured with different wavelengths does not share the same scale for better readability. S39

S1. Experimental part

In this supplementary section, a detail of colloids preparation and subsequent modification will be described. Values of the concentration, referred in the text, are in all cases referring to the value of initial concentration. Concentration of nanoparticles was calculated using standard procedure, which is possible due to the fact that both types of nanoparticles used show a spherical morphology.[1,2] Briefly, a whole volume of metal added to reaction mixture is calculated at the first step. Subsequently, this value is divided by the volume of one hypothetical particle. This process assumes that all particles have a diameter equal to the mean diameter obtained from EM analysis. Therefore, it should be kept in mind that real concentration may differ to some extent, whereas colloids exhibit wider distribution of diameters.

Gold nanoparticles

Table S1. Reactants and their concentrations and volumes used for the gold colloids preparation and subsequent modification

Substance	Initial concentration (mol/L)	Added volume (mL)	Final concentration in reaction mixture (mol/L)
HA·HCl	$2.88 \cdot 10^{-3}$	100.0	$2.84 \cdot 10^{-3}$
$HAuCl_4$	$5.89 \cdot 10^{-2}$	0.5	$2.90 \cdot 10^{-4}$
NaOH	$2.53 \cdot 10^{-1}$	1.0	$2.49 \cdot 10^{-3}$
	Total volume (mL)	101.5	
AuNPs	$4.69 \cdot 10^{-9}$	4.5	$4.22 \cdot 10^{-9}$
$Analyte_{(a)/(b)/(c)}$	$1.00 \cdot 10^{-2}\ _{(a)}$ / $1.00 \cdot 10^{-3}\ _{(b)}$ / $1.00 \cdot 10^{-4}\ _{(c)}$	0.5	$1.00 \cdot 10^{-3}\ _{(a)}$ / $1.00 \cdot 10^{-4}\ _{(b)}$ / $1.00 \cdot 10^{-5}\ _{(c)}$
	Total volume (mL)	5.0	

Gold nanoparticles were prepared according to the preparation of I. S. Todór et al.[1] In an Erlenmeyer flask, 100 mL of hydroxylamine hydrochloride solution (HA·HCl) with the concentration of $2.88 \cdot 10^{-3}$ mol/L was mixed with 0.5 ml of $HAuCl_4$ with concentration of $5.89 \cdot 10^{-2}$ mol/L. This mixture was stirred for 5 min at room temperature at 250 rpm. Subsequently, 1 mL of NaOH solution ($2.53 \cdot 10^{-1}$ mol/L) was added (substances‘ concentrations in the reaction mixture are listed in Table S1). As a result, red colloid containing spherical nanoparticles with a mean diameter of 20 nm,[1] was obtained. 4.5 ml of this colloid (with concentration of $4.69 \cdot 10^{-9}$ mol/L) was modified by the 0.5 ml of analyte (AMP, MET or MDMA) with the concentration of $1.00 \cdot 10^{-2}$, $1.00 \cdot 10^{-3}$ or $1.00 \cdot 10^{-4}$ mol/L, depending on the final desired concentration of analyte in the solution ($1.00 \cdot 10^{-3}$, $1.00 \cdot 10^{-4}$ or $1.00 \cdot 10^{-5}$ mol/L).

Silver nanoparticles

Table S2. Reactants and their concentrations and volumes used for the silver colloids preparation and subsequent modification

Substance	Initial concentration (mol/L)	Added volume (mL)	Final concentration in reaction mixture (mol/L)
HA·HCl	$3.71 \cdot 10^{-3}$	22.5	$1.67 \cdot 10^{-3}$
NaOH	$7.40 \cdot 10^{-3}$	22.5	$3.33 \cdot 10^{-3}$
$AgNO_3$	$1.00 \cdot 10^{-2}$	5.0	$1.00 \cdot 10^{-3}$
	Total volume (mL)	50.0	
AgNPs	$1.07 \cdot 10^{-10}$	4.5	$9.65 \cdot 10^{-11}$
$\text{Analyte}_{(a)/(b)/(c)}$	$1.00 \cdot 10^{-2}$ (a) / $1.00 \cdot 10^{-3}$ (b) / $1.00 \cdot 10^{-4}$ (c)	0.5	$1.00 \cdot 10^{-3}$ (a) / $1.00 \cdot 10^{-4}$ (b) / $1.00 \cdot 10^{-5}$ (c)
	Total volume (mL)	5.0	

Silver colloidal solutions of nanoparticles (AgNPs) were prepared according to the preparation of authors N. Leopold and B. Lendl.[2] A 22.5 ml of NaOH solution with a concentration of $7.40 \cdot 10^{-3}$ mol/L was mixed with a solution of hydroxylamine hydrochloride with a volume of 22.5 mL and a concentration of $3.71 \cdot 10^{-3}$ mol/L. Next, $AgNO_3$ solution with a volume of 5 mL and a concentration of 10^{-2} mol/L was added to the mixture with rapid mixing (substances‘ concentrations in the reaction mixture are listed in Table S2). After 10 min, a yellowish solution of silver nanoparticles with a spherical morphology and a mean diameter of 23 nm is formed.[2] 4.5 ml of this colloid (with concentration of $1.07 \cdot 10^{-10}$ mol/L) was modified by the 0.5 ml of analyte (AMP, MET or MDMA) with concentration of $1.00 \cdot 10^{-2}$, $1.00 \cdot 10^{-3}$ or $1.00 \cdot 10^{-4}$ mol/L, depending on the final desired concentration of analyte in solution ($1.00 \cdot 10^{-3}$, $1.00 \cdot 10^{-4}$ or $1.00 \cdot 10^{-5}$ mol/L).

S2. Amphetamine

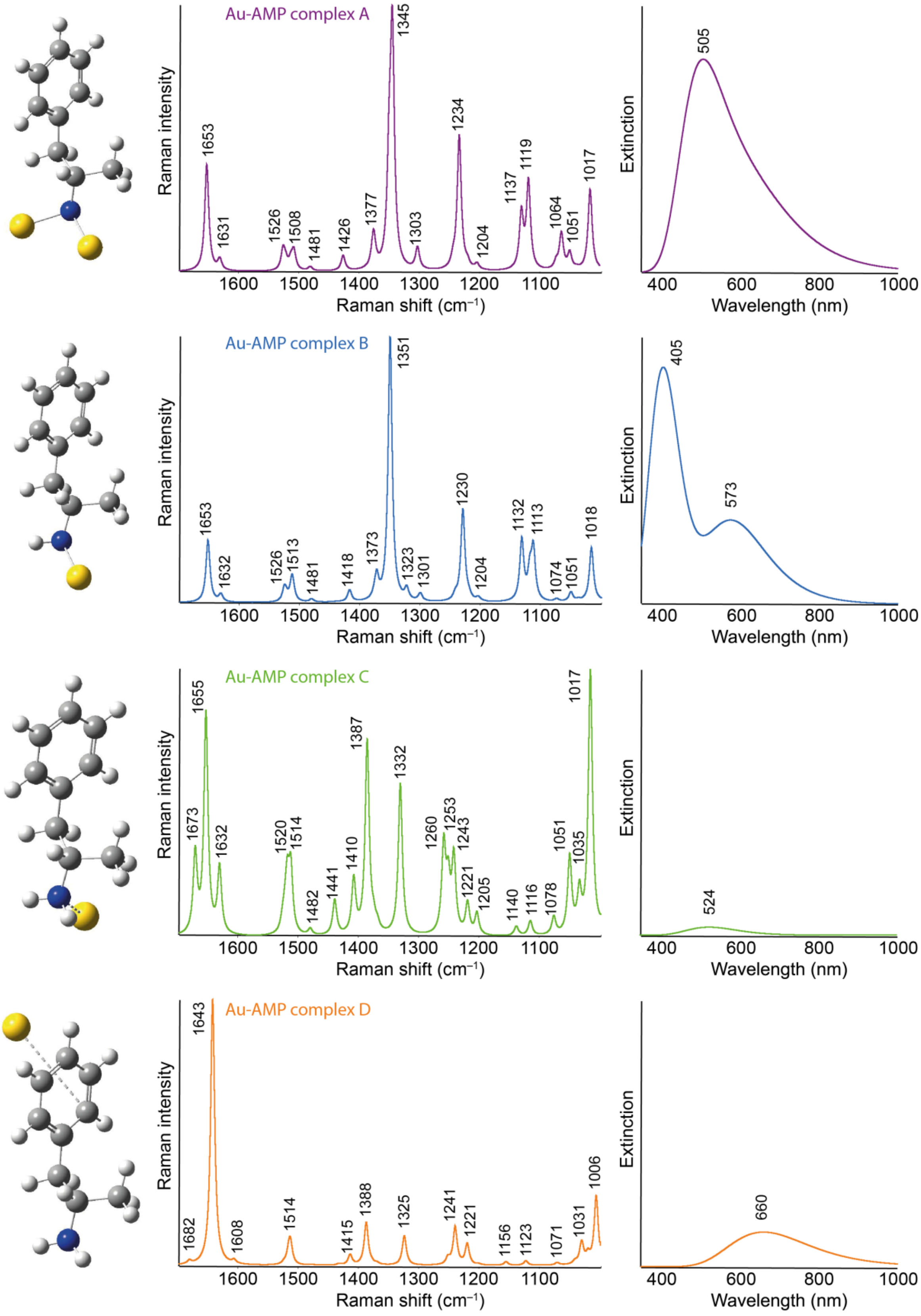

Figure S1. Structure, DFT Raman and UV-Vis spectra of the considered Au-AMP complexes.

Table S3. Assignment of the vibrational modes of DFT spectra of Au–AMP complex A

Raman shift (cm^{-1})		
DFT	DFT (scaled 0.97)	Assignment of the vibrational modes
1653	1603	ν $(C–C)_{ar}$
1631	1582	ν $(C=C)_{ar}$
1526	1480	ν $(C–C)_{ar}$
1508	1463	δ_{sci} $(–CH_2)$, δ $(–CH)_{ar}$
1481	1437	δ_{twi} $(–CH_2)$, δ $(–CH)_{ar}$
1426	1384	δ_{umb} $(–CH_3)$
1377	1336	δ_{twi} $(–CH_2)$, δ $(–CH)_{ar}$
1345	1304	δ_{wag} $(–CH_2)$, δ (–CH), δ $(–CH)_{ar}$
1303	1264	δ_{wag} $(–CH_2)$, δ (–CH)
1234	1207	δ_{twi} $(–CH_2)$, δ (–CH), δ $(–CH)_{ar}$
1220	1184	δ_{ip} $(–CH)_{ar}$
1204	1168	δ_{ip} $(–CH)_{ar}$
1131	1097	δ_{twi} $(–CH_2)$, δ (–CH), δ $(–CH_3)$, δ $(–CH)_{ar}$
1119	1085	δ_{twi} $(–CH_2)$, δ (–CH), δ $(–CH_3)$, δ $(–CH)_{ar}$
1073	1041	δ_{twi} $(–CH_2)$, δ (–CH), δ $(–CH_3)$, δ $(–CH)_{ar}$
1064	1032	δ $(–CH_3)$, δ $(–CH)_{aryl}$
1051	1019	δ_{ip} $(–CH)_{ar}$
1017	986	δ_{oop} $(–CH)_{ar}$

ν – stretching, δ – deformation, sci – scissoring, twi – twisting, wag – wagging, roc – rocking vibration, ar – aromatic, ip – in-plane, oop – out of-plane

Table S4. Assignment of the vibrational modes of DFT spectra of Au–AMP complex B

Raman shift (cm^{-1})		
DFT	DFT (scaled 0.97)	Assignment of the vibrational modes
1653	1603	ν (C–C)$_{ar}$
1632	1583	ν (C=C)$_{ar}$
1526	1480	δ_{sci} (–CH_2), δ (–CH_3), δ (–CH)$_{ar}$
1513	1467	δ_{sci} (–CH_2), δ_{sci} (–CH_3),
1481	1437	δ_{twi} (–CH_2), δ (–CH)$_{ar}$
1418	1375	δ_{umb} (–CH_3), δ (–CH), δ_{twi} (–CH_2), δ (–NH)
1373	1332	δ (–CH), δ (–CH_2), δ (–CH)$_{ar}$
1351	1310	δ (–CH), δ_{wag} (–CH_2)
1323	1283	δ (–CH), δ_{wag} (–CH_2)
1301	1262	δ (–NH), δ (–CH), δ_{wag} (–CH_2), δ (–CH_3)
1243	1205	skeletal
1230	1193	skeletal
1204	1168	δ_{ip} (–CH)$_{ar}$
1132	1098	skeletal
1119	1085	skeletal
1113	1080	ν (N–C), δ (–CH_2), δ (–CH_3), δ (–CH)$_{ar}$
1074	1042	δ (–CH_2), δ (–CH_3), δ (–CH)$_{ar}$
1051	1019	δ_{ip} (–CH)$_{ar}$
1018	987	δ_{oop} (–CH)$_{ar}$

ν – stretching, δ – deformation, sci – scissoring, twi – twisting, wag – wagging, roc – rocking vibration, ar – aromatic, ip – in-plane, oop – out of-plane

Table S5. Assignment of the vibrational modes of DFT spectra of Au–AMP complex C

Raman shift (cm^{-1})		
DFT	DFT (scaled 0.97)	Assignment of the vibrational modes
1673	1622	δ_{sci} (–NH_2)
1655	1605	ν (C=C)$_{ar}$
1632	1583	ν (C–C)$_{ar}$, δ_{twi} (–CH_2)
1520	1474	δ_{sci} (–CH_3), δ_{sci} (–CH_2)
1514	1469	δ_{sci} (–CH_2), δ (–CH_3)
1482	1437	δ_{twi} (–CH_2), δ (–CH)$_{ar}$
1441	1398	δ_{umb} (–CH_3)
1410	1367	δ_{twi} (–NH_2), δ_{twi} (–CH_2), δ (–CH), δ (–CH_3)
1387	1346	δ (–CH), δ_{wag} (–CH_2)
1332	1292	δ (–CH), δ_{wag} (–CH_2)
1260	1222	δ_{twi} (–NH_2), δ_{twi} (–CH_2), δ (–CH_3)
1253	1215	δ_{twi} (–NH_2), δ_{twi} (–CH_2), δ (–CH_3)
1243	1206	ν (C–C), δ_{ip} (–CH)$_{ar}$
1221	1184	δ_{ip} (–CH)$_{ar}$
1205	1169	δ_{ip} (–CH)$_{ar}$
1140	1105	skeletal
1116	1083	skeletal
1078	1045	skeletal
1051	1020	δ_{ip} (–CH)$_{ar}$
1035	1004	δ_{twi} (–NH_2), δ_{oop} (–CH)$_{ar}$
1017	986	$\delta_{ip, br}$ (–CH)$_{ar}$

ν – stretching, δ – deformation, sci – scissoring, twi – twisting, wag – wagging, roc – rocking vibration, ar – aromatic, ip – in-plane, oop – out of-plane

Table S6. Assignment of the vibrational modes of DFT spectra of Au–AMP complex D

Raman shift (cm^{-1})		
DFT	DFT (scaled 0.97)	Assignment of the vibrational modes
1682	1631	δ_{sci} (–NH_2)
1643	1594	ν (C=C)$_{ar}$
1608	1560	ν (C–C)$_{ar}$
1514	1469	δ_{sci} (–CH_2), δ (–CH)$_{ar}$
1433	1390	δ_{umb} (–CH_3)
1415	1372	δ (–CH), δ_{twi} (–CH_2), δ_{roc} (–NH_2)
1388	1347	δ (–CH), δ_{wag} (–CH_2)
1325	1285	δ (–CH), δ_{wag} (–CH_2)
1252	1214	δ_{twi} (–NH_2), δ (–CH_3), δ (–CH)
1241	1203	δ (–CH_2), δ (–CH)$_{ar}$, ν (C–C)$_{aryl}$
1221	1184	δ_{ip} (–CH)$_{ar}$
1201	1165	δ_{ip} (–CH)$_{ar}$
1156	1122	δ_{twi} (–CH_2), δ (–CH), δ (–CH_3), δ (–NH_2)
1123	1090	skeletal
1071	1039	skeletal
1031	1000	δ_{oop} (–CH)$_{ar}$
1020	990	δ (–CH)$_{ar}$
1006	976	δ (–CH_3), δ (–NH_2)
953	924	δ_{oop} (–CH)$_{ar}$, δ (–CH_3)

ν – stretching, δ – deformation, sci – scissoring, twi – twisting, wag – wagging, roc – rocking vibration, ar – aromatic, ip – in-plane, oop – out of-plane

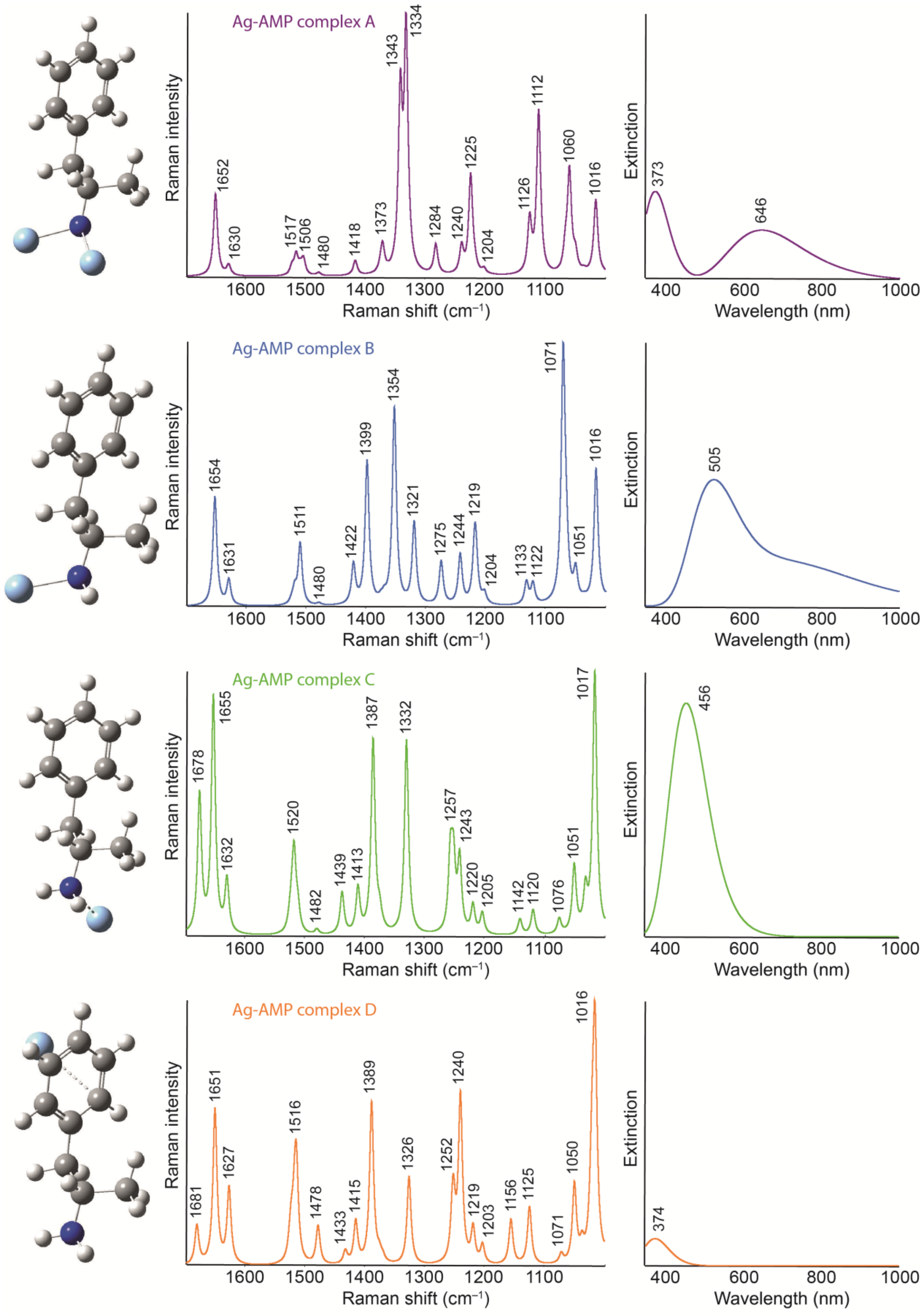

Figure S2. Structure, DFT Raman and UV-Vis spectra of the considered Ag-AMP complexes.

Table S7. Assignment of the vibrational modes of DFT spectra of Ag–AMP complex A

Raman shift (cm^{-1})		
DFT	DFT (scaled 0.97)	Assignment of the vibrational modes
1652	1603	ν (C–C)$_{ar}$
1630	1581	ν (C=C)$_{ar}$
1517	1472	δ_{sci} (–CH_2), δ_{sci} (–CH_3)
1505	1460	δ_{sci} (–CH_2), δ_{sci} (–CH_3)
1480	1435	δ_{ip} (–CH)$_{ar}$, δ_{twi} (–CH_2)
1418	1376	δ_{umb} (–CH_3)
1373	1332	δ_{ip} (–CH)$_{ar}$, δ_{twi} (–CH_2), δ (–CH)
1343	1303	δ (–CH), δ_{twi} (–CH_2)
1333	1293	δ (–CH), δ_{wag} (–CH_2)
1284	1245	δ (–CH), δ_{wag} (–CH_2)
1241	1203	ν (C–C), δ_{wag} (–CH_2), δ_{ip} (–CH)$_{ar}$
1225	1189	δ (–CH), δ_{twi} (–CH_2), δ (–CH_3)
1203	1167	δ_{ip} (–CH)$_{ar}$
1127	1093	skeletal
1112	1078	skeletal
1060	1028	ν (C–N), δ (–CH), δ (–CH_3), δ_{oop} (–CH)$_{ar}$
1016	986	δ_{oop} (–CH)$_{ar}$

ν – stretching, δ – deformation, sci – scissoring, twi – twisting, wag – wagging, roc – rocking vibration, ar – aromatic, ip – in-plane, oop – out of-plane

Table S8. Assignment of the vibrational modes of DFT spectra of Ag–AMP complex B

Raman shift (cm^{-1})		
DFT	DFT (scaled 0.97)	Assignment of the vibrational modes
1654	1604	ν (C–C)$_{ar}$
1631	1582	ν (C=C)$_{ar}$
1511	1466	δ (–CH_3), $δ_{sci}$ (–CH_2)
1480	1436	$δ_{twi}$ (–CH), $δ_{ip}$ (–CH)$_{ar}$
1422	1379	$δ_{umb}$ (–CH_3)
1399	1357	δ (–CH_2), δ (–CH), $δ_{umb}$ (–CH_3), δ (–NH)
1354	1313	$δ_{wag}$ (–CH_2), δ (–CH), δ (–CH_3)
1321	1281	$δ_{wag}$ (–CH_2), δ (–CH), δ (–NH)
1275	1237	$δ_{wag}$ (–CH_2), δ (–CH), δ (–NH)
1244	1206	ν (C–C), $δ_{wag}$ (–CH_2)
1220	1183	$δ_{twi}$ (–CH_2), $δ_{ip}$ (–CH)$_{ar}$, δ (–CH), δ (–NH)
1203	1167	$δ_{ip}$ (–CH)$_{ar}$
1133	1099	skeletal vibration
1122	1088	skeletal vibration
1071	1039	skeletal vibration
1050	1019	$δ_{ip}$ (–CH)$_{ar}$
1016	986	$δ_{oop}$ (–CH)$_{ar}$

ν – stretching, δ – deformation, sci – scissoring, twi – twisting, wag – wagging, roc – rocking vibration, ar – aromatic, ip – in-plane, oop – out of-plane

Table S9. Assignment of the vibrational modes of DFT spectra of Ag–AMP complex C

Raman shift (cm^{-1})		
DFT	DFT (scaled 0.97)	Assignment of the vibrational modes
1678	1627	δ_{sci} (–NH_2)
1655	1605	ν (C–C)$_{ar}$
1632	1583	ν (C=C)$_{ar}$, δ_{twi} (–CH_2)
1520	1474	δ_{sci} (–CH_3)
1482	1437	δ_{twi} (–CH_2), δ_{ip} (–CH)$_{ar}$
1439	1396	δ_{umb} (–CH_3)
1413	1370	δ_{twi} (–NH_2), δ_{twi} (–CH_2), δ (–CH), δ (–CH_3)
1388	1346	δ_{wag} (–CH_2), δ (–CH), δ (–CH)$_{ar}$
1332	1292	δ_{wag} (–CH_2), δ (–CH)
1258	1221	δ_{twi} (–NH_2), δ_{twi} (–CH_2), δ (–CH), δ (–CH_3)
1253	1216	δ_{twi} (–NH_2), δ_{twi} (–CH_2), δ (–CH), δ (–CH_3)
1243	1205	ν (C–C), δ_{wag} (–CH_2), δ (–CH)$_{ar}$
1220	1183	δ_{ip} (–CH)$_{ar}$
1205	1169	δ_{ip} (–CH)$_{ar}$
1142	1108	skeletal vibration
1120	1086	skeletal vibration
1076	1044	skeletal vibration
1051	1020	δ_{ip} (–CH)$_{ar}$
1032	1001	δ_{twi} (–NH_2), ν (C–C), δ (–CH_3)
1017	986	δ_{oop} (–CH)$_{ar}$

ν – stretching, δ – deformation, sci – scissoring, twi – twisting, wag – wagging, roc – rocking vibration, ar – aromatic, ip – in-plane, oop – out of-plane

Table S10. Assignment of the vibrational modes of DFT spectra of Ag–AMP complex D

Raman shift (cm^{-1})		
DFT	DFT (scaled 0.97)	Assignment of the vibrational modes
1681	1631	δ_{sci} (–NH_2)
1651	1601	ν (C–C)$_{ar}$
1627	1579	ν (C=C)$_{ar}$, δ_{twi} (–CH_2)
1515	1469	δ_{sci} (–CH_2), δ_{sci} (–CH_3)
1478	1434	δ_{twi} (–CH_2), δ_{ip} (–CH)$_{ar}$
1433	1390	δ_{umb} (–CH_3)
1415	1373	δ_{roc} (–NH_2), δ (–CH), δ_{twi} (–CH_2), δ (–CH_3)
1389	1347	δ (–CH), δ_{wag} (–CH_2), δ (–CH_3)
1326	1287	δ_{twi} (–NH_2), δ (–CH)
1253	1215	δ_{twi} (–NH_2), δ_{twi} (–CH_2), δ (–CH_3), δ (–CH)
1240	1203	ν (C–C), δ (–CH_2), δ_{ip} (–CH)$_{ar}$
1219	1183	δ_{ip} (–CH)$_{ar}$
1203	1167	δ_{ip} (–CH)$_{ar}$
1156	1121	skeletal
1125	1091	skeletal
1072	1040	skeletal
1050	1018	δ_{ip} (–CH)$_{ar}$
1037	1006	δ_{oop} (–CH)$_{ar}$
1017	986	δ_{oop} (–CH)$_{ar}$

ν – stretching, δ – deformation, sci – scissoring, twi – twisting, wag – wagging, roc – rocking vibration, ar – aromatic, ip – in-plane, oop – out of-plane

S3. Methamphetamine

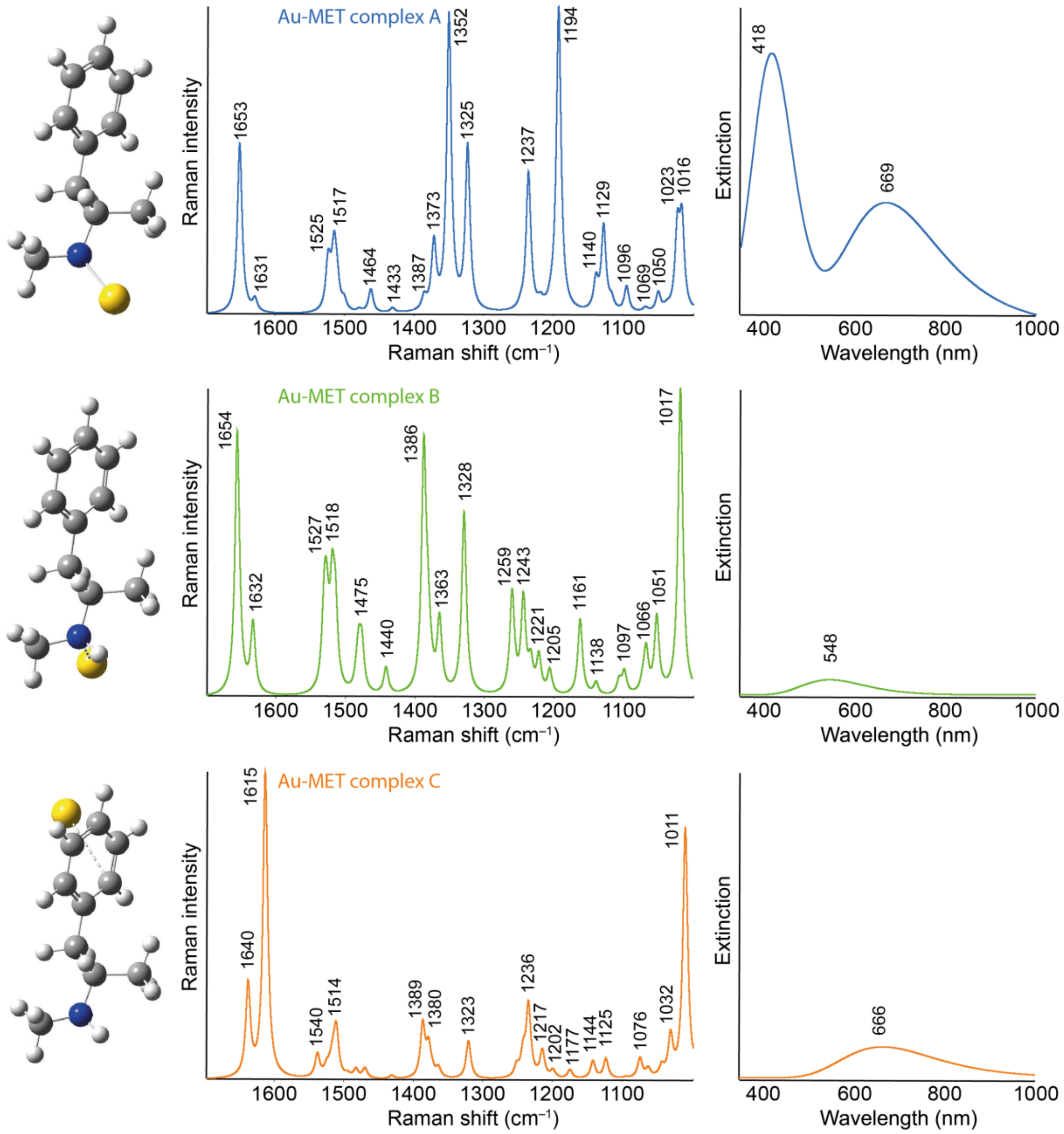

Figure S3. Structure, DFT Raman and UV-Vis spectra of the considered Au-MET complexes.

Table S11. Assignment of the vibrational modes of DFT spectra of Au–MET complex A

Raman shift (cm^{-1})		
DFT	DFT (scaled 0.97)	Assignment of the vibrational modes
1653	1603	ν (C–C)$_{ar}$
1631	1582	ν (C=C)$_{ar}$, δ_{twi} (–CH_2)
1526	1480	δ (–CH_3)$_N$, δ_{sci} (–CH_2), δ (–CH_3)$_C$, δ (–CH)$_{ar}$
1517	1472	δ (–CH_3)$_N$, δ (–CH_3)$_C$
1481	1436	δ_{twi} (–CH_2), δ (–CH)$_{ar}$
1464	1420	δ_{umb} (–CH_3)$_N$
1433	1390	δ_{umb} (–CH_3)$_C$
1387	1 346	δ_{twi} (–CH_2), δ (–CH)
1373	1332	δ_{twi} (–CH_2), δ (–CH), δ_{ip} (–CH)$_{ar}$
1352	1311	δ_{twi} (–CH_2), δ (–CH), δ_{ip} (–CH)$_{ar}$
1325	1285	δ_{wag} (–CH_2), δ (–CH)
1237	1200	skeletal vibration
1220	1183	δ_{ip} (–CH)$_{ar}$
1194	1158	ν (C–N), δ (–CH_3)$_{N,C}$, δ_{twi} (–CH_2)
1140	1106	δ (–CH)$_{aryl}$
1129	1095	δ_{twi} (–CH_2), δ (–CH_3)$_C$, δ (–CH)$_{ar}$
1096	1063	δ (–CH_3)$_N$, δ_{twi} (–CH_2), δ (–CH_3)$_C$, δ (–CH)$_{ar}$
1069	1037	δ (–CH_3)$_{N,C}$, δ_{twi} (–CH_2), δ (–CH), δ (–CH)$_{ar}$
1050	1019	δ_{ip} (–CH)$_{ar}$
1023	992	ν (C–N), δ_{oop} (–CH)$_{ar}$
1016	986	δ_{oop} (–CH)$_{ar}$

ν – stretching, δ – deformation, sci – scissoring, twi – twisting, wag – wagging, roc – rocking vibration, ar – aromatic, ip – in-plane, oop – out of-plane

Table S12. Assignment of the vibrational modes of DFT spectra of Au–MET complex B

Raman shift (cm^{-1})		
DFT	DFT (scaled 0.97)	Assignment of the vibrational modes
1654	1605	ν (C–C)$_{ar}$
1632	1583	ν (C=C)$_{ar}$, $δ_{twi}$ (–CH_2)
1527	1481	δ (–NH), $δ_{sci}$ (–CH_3)$_{N,C}$, $δ_{sci}$ (–CH_2)
1518	1472	δ (–NH), $δ_{sci}$ (–CH_3)$_{N,C}$, $δ_{sci}$ (–CH_2)
1475	1431	$δ_{umb}$ (–CH_3)$_N$
1440	1397	$δ_{umb}$ (–CH_3)$_C$
1386	1344	$δ_{wag}$ (–CH_2), δ (–CH)
1363	1 322	$δ_{roc}$ (–CH_2), δ (–CH), δ (–CH)$_{ar}$
1328	1288	$δ_{wag}$ (–CH_2), δ (–CH)
1259	1221	δ (–CH_3)$_{N,C}$, $δ_{twi}$ (–CH_2), δ (–CH)
1243	1206	ν (C–C), δ (–CH_2), δ (–CH)$_{ar}$
1232	1195	ν (C–C), δ (–CH_3)$_{N,C}$, δ (–CH)
1221	1184	$δ_{ip}$ (–CH)$_{ar}$
1205	1169	$δ_{ip}$ (–CH)$_{ar}$
1161	1126	ν (C–N), δ (–CH_3)$_N$, δ (–NH)
1138	1104	skeletal vibration
1097	1065	ν (C–N), δ (–NH), δ (–CH_3)$_{N,C}$
1066	1034	ν (C–N), δ (–CH_3)$_{C,}$ ν (C–C), δ (–NH)
1051	1019	$δ_{ip,br}$ (–CH)$_{ar}$
1017	986	$δ_{oop}$ (–CH)$_{ar}$

ν – stretching, δ – deformation, sci – scissoring, twi – twisting, wag – wagging, roc – rocking vibration, ar – aromatic, ip – in-plane, oop – out of-plane

Table S13. Assignment of the vibrational modes of DFT spectra of Au–MET complex C

Raman shift (cm^{-1})		
DFT	DFT (scaled 0.97)	Assignment of the vibrational modes
1640	1591	ν $(C–C)_{ar}$
1615	1567	ν $(C=C)_{ar}$
1540	1494	$δ_{sci}$ $(–CH_3)_N$
1389	1347	$δ_{wag}$ $(–CH_2)$, δ (–CH)
1380	1339	$δ_{twi}$ $(–CH_2)$, δ (–CH), δ $(–CH)_{ar}$, δ (–NH)
1323	1283	$δ_{wag}$ $(–CH_2)$, δ (–CH)
1254	1216	$δ_{twi}$ $(–CH_2)$, δ $(–CH)_{ar}$, δ $(–CH_3)_C$
1244, 1236	1207, 1199	δ $(–CH_3)_{C,N}$, $δ_{twi}$ $(–CH_2)$, δ (–CH), δ $(–CH)_{ar}$, ν (C–C), ν (C–N)
1217	1180	$δ_{ip}$ $(–CH)_{ar}$
1201	1165	$δ_{ip}$ $(–CH)_{ar}$
1177	1141	ν (C–N), $δ_{twi}$ $(–CH_2)$, δ (–CH), δ $(–CH_3)_N$
1144	1109	$δ_{twi}$ $(–CH_2)$, δ (–CH), δ $(–CH_3)_N$
1125	1091	skeletal vibration
1045	1013	δ $(–CH)_{ar}$, ν $(C–C)_{ar}$, ν $(C=C)_{ar}$
1032	1001	$δ_{oop}$ $(–CH)_{ar}$
1011	980	$δ_{oop}$ $(–CH)_{ar}$
956	927	$δ_{oop}$ $(–CH)_{ar}$, δ $(–CH_3)_C$
937	909	δ $(–CH_3)_C$, ν (C–N)

ν – stretching, δ – deformation, sci – scissoring, twi – twisting, wag – wagging, roc – rocking vibration, ar – aromatic, ip – in-plane, oop – out of-plane

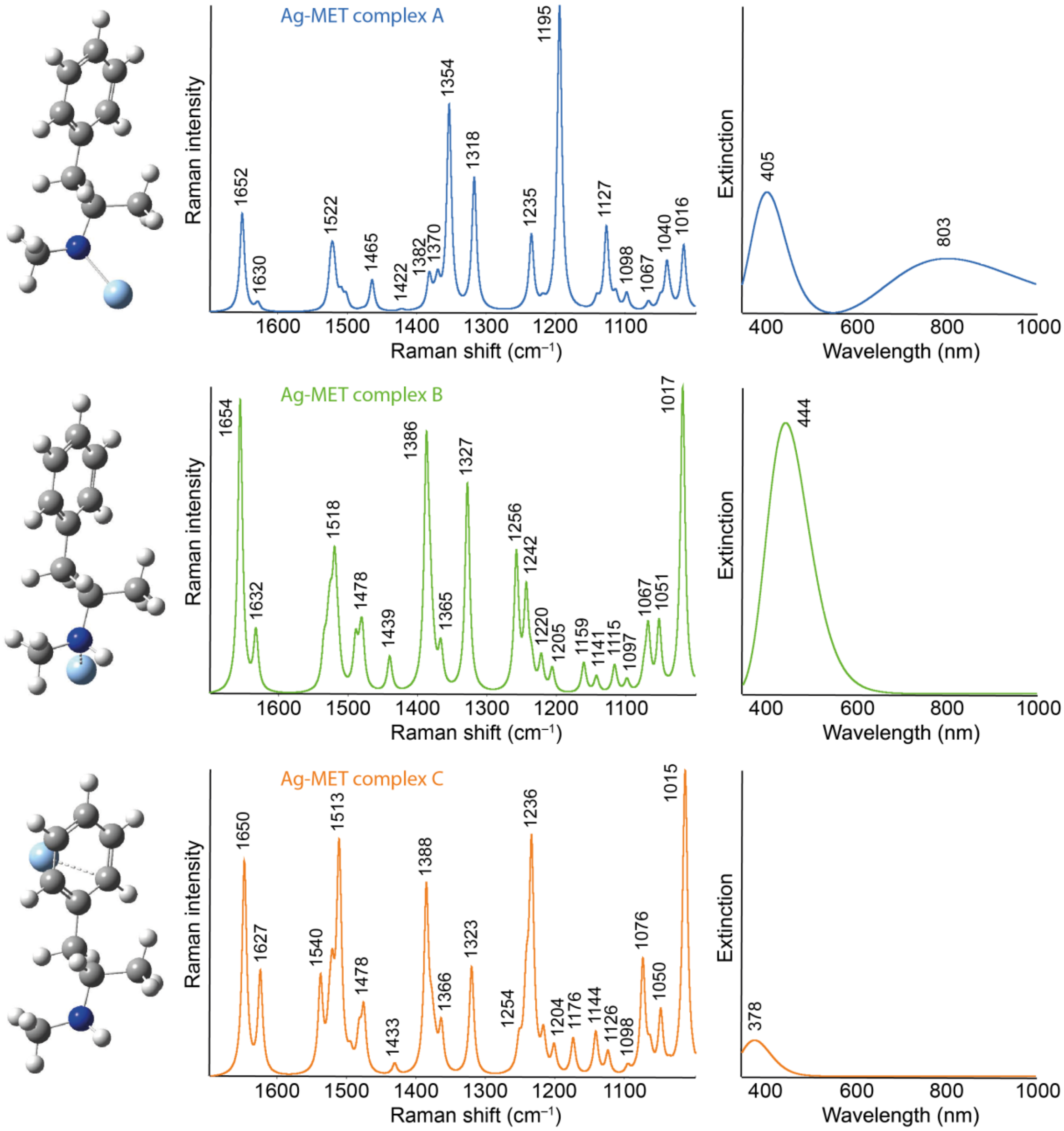

Figure S4. Structure, DFT Raman and UV-Vis spectra of the considered Ag-MET complexes.

Table S14. Assignment of the vibrational modes of DFT spectra of Ag–MET complex A

Raman shift (cm^{-1})		
DFT	DFT (scaled 0.98)	Assignment of the vibrational modes
1652	1619	ν (C–C)$_{ar}$
1630	1597	ν (C=C)$_{ar}$, δ_{twi} (–CH_2)
1522	1491	δ (–CH_3)$_N$, δ_{sci} (–CH_2), δ_{ip} (–CH)$_{ar}$
1509	1479	δ (–CH_3)$_{N,C}$, δ_{sci} (–CH_2)
1465	1436	δ_{umb} (–CH_3)$_N$
1422	1393	δ_{umb} (–CH_3)$_C$
1382	1355	δ (–CH), δ_{twi} (–CH_2), δ (–CH_3)$_C$, δ_{ip} (–CH)$_{ar}$
1370	1343	δ (–CH), δ_{twi} (–CH_2), δ (–CH_3)$_{C,N}$, δ_{ip} (–CH)$_{ar}$
1354	1327	δ (–CH), δ_{wag} (–CH_2), δ_{ip} (–CH)$_{ar}$
1318	1291	δ (–CH), δ_{wag} (–CH_2)
1235	1211	ν (C–C), δ_{twi} (–CH_2), δ (–CH_3)$_{N,C}$, δ (–CH)
1219	1195	δ_{ip} (–CH)$_{ar}$
1195	1171	ν (C–N), δ_{twi} (–CH_2), δ (–CH_3)$_{N,C}$, δ (–CH)
1141	1118	skeletal
1127	1105	skeletal
1114	1092	δ (–CH_3)$_{N,C}$, δ (–CH)
1098	1076	skeletal
1067	1045	skeletal
1040	1019	ν (C–N), δ (–CH_2), δ (–CH_3)$_{N,C}$, δ (–CH), δ_{oop} (–CH)$_{ar}$
1016	995	δ_{oop} (–CH)$_{ar}$

ν – stretching, δ – deformation, sci – scissoring, twi – twisting, wag – wagging, roc – rocking vibration, ar – aromatic, ip – in-plane, oop – out of-plane

Table S15. Assignment of the vibrational modes of DFT spectra of Ag–MET complex B

Raman shift (cm^{-1})		
DFT	DFT (scaled 0.98)	Assignment of the vibrational modes
1654	1621	ν $(C–C)_{ar}$
1632	1599	ν $(C=C)_{ar}$, $δ_{twi}$ $(–CH_2)$
1518	1488	$δ_{sci}$ $(–CH_2)$, δ $(–CH_3)_{N,C}$, δ (–NH)
1488	1458	$δ_{sci}$ $(–CH_2)$, δ $(–CH_3)_{N,C}$, δ (–NH), δ (–CH)
1478	1449	$δ_{umb}$ $(–CH_3)_N$
1439	1410	$δ_{umb}$ $(–CH_3)_C$
1386	1359	$δ_{wag}$ $(–CH_2)$, δ (–CH)
1365	1338	δ (–NH), δ (–CH), $δ_{ip}$ $(–CH)_{ar}$
1327	1300	$δ_{wag}$ $(–CH_2)$, δ (–CH)
1256	1231	$δ_{twi}$ $(–CH_2)$, δ $(–CH_3)_{N,C}$, δ (–CH), $δ_{ip}$ $(–CH)_{ar}$
1242	1217	ν (C–C), $δ_{twi}$ $(–CH_2)$, δ $(–CH_3)_{N,C}$, δ (–CH), $δ_{ip}$ $(–CH)_{ar}$
1220	1196	$δ_{ip}$ $(–CH)_{ar}$
1205	1181	$δ_{ip}$ $(–CH)_{ar}$
1159	1136	ν (C–N), δ $(–CH_3)_N$
1141	1118	skeletal
1115	1093	skeletal
1097	1075	δ $(–CH)_{aryl}$
1067	1045	δ $(–CH)_{aryl}$
1051	1030	$δ_{ip}$ $(–CH)_{ar}$
1017	996	$δ_{oop}$ $(–CH)_{ar}$

ν – stretching, δ – deformation, sci – scissoring, twi – twisting, wag – wagging, roc – rocking vibration, ar – aromatic, ip – in-plane, oop – out of-plane

Table S16. Assignment of the vibrational modes of DFT spectra of Ag–MET complex C

Raman shift (cm^{-1})		
DFT	DFT (scaled 0.97)	Assignment of the vibrational modes
1650	1617	ν $(C–C)_{ar}$
1627	1594	ν $(C=C)_{ar}$, $δ_{twi}$ $(–CH_2)$
1540	1509	δ $(–CH_3)_N$, δ (–NH)
1524	1493	δ $(–CH_3)_C$, $δ_{ip}$ $(–CH)_{ar}$
1513	1483	δ $(–CH_3)_{N,C}$, δ (–NH), $δ_{sci}$ $(–CH_2)$
1497	1467	δ $(–CH_3)_{N,C}$, δ (–NH), $δ_{twi}$ $(–CH_2)$
1478	1448	$δ_{twi}$ $(–CH_2)$, $δ_{ip}$ $(–CH_3)_{ar}$
1433	1405	$δ_{umb}$ $(–CH_3)_C$
1388	1360	$δ_{wag}$ $(–CH_2)$, δ (–CH)
1366	1339	$δ_{wag}$ $(–CH_2)$, δ (–CH), δ (–NH), $δ_{ip}$ $(–CH)_{ar}$
1323	1296	$δ_{wag}$ $(–CH_2)$, δ (–CH)
1254	1229	$δ_{twi}$ $(–CH_2)$, δ $(–CH_3)_{N,C}$, δ (–CH), $δ_{ip}$ $(–CH)_{ar}$
1236	1211	ν (C–C), $δ_{twi}$ $(–CH_2)$, δ $(–CH_3)_{N,C}$, δ (–CH), $δ_{ip}$ $(–CH)_{ar}$
1219	1195	$δ_{ip}$ $(–CH)_{ar}$
1204	1180	$δ_{ip}$ $(–CH)_{ar}$
1176	1153	ν (C–N), δ $(–CH_3)_N$, $δ_{twi}$ $(–CH_2)$
1144	1121	skeletal
1126	1104	skeletal
1098	1076	skeletal
1076	1054	ν (C–C), $δ_{twi}$ $(–CH_2)$, δ $(–CH_3)_C$, $δ_{ip}$ $(–CH)_{ar}$
1065	1044	$δ_{twi}$ $(–CH_2)$, δ $(–CH_3)_{N,C}$, δ (–CH), $δ_{ip}$ $(–CH)_{ar}$
1050	1029	$δ_{ip}$ $(–CH)_{ar}$
1015	994	$δ_{oop}$ $(–CH)_{ar}$

ν – stretching, δ – deformation, sci – scissoring, twi – twisting, wag – wagging, roc – rocking vibration, ar – aromatic, ip – in-plane, oop – out of-plane

S4. Methylenedioxymethamphetamine

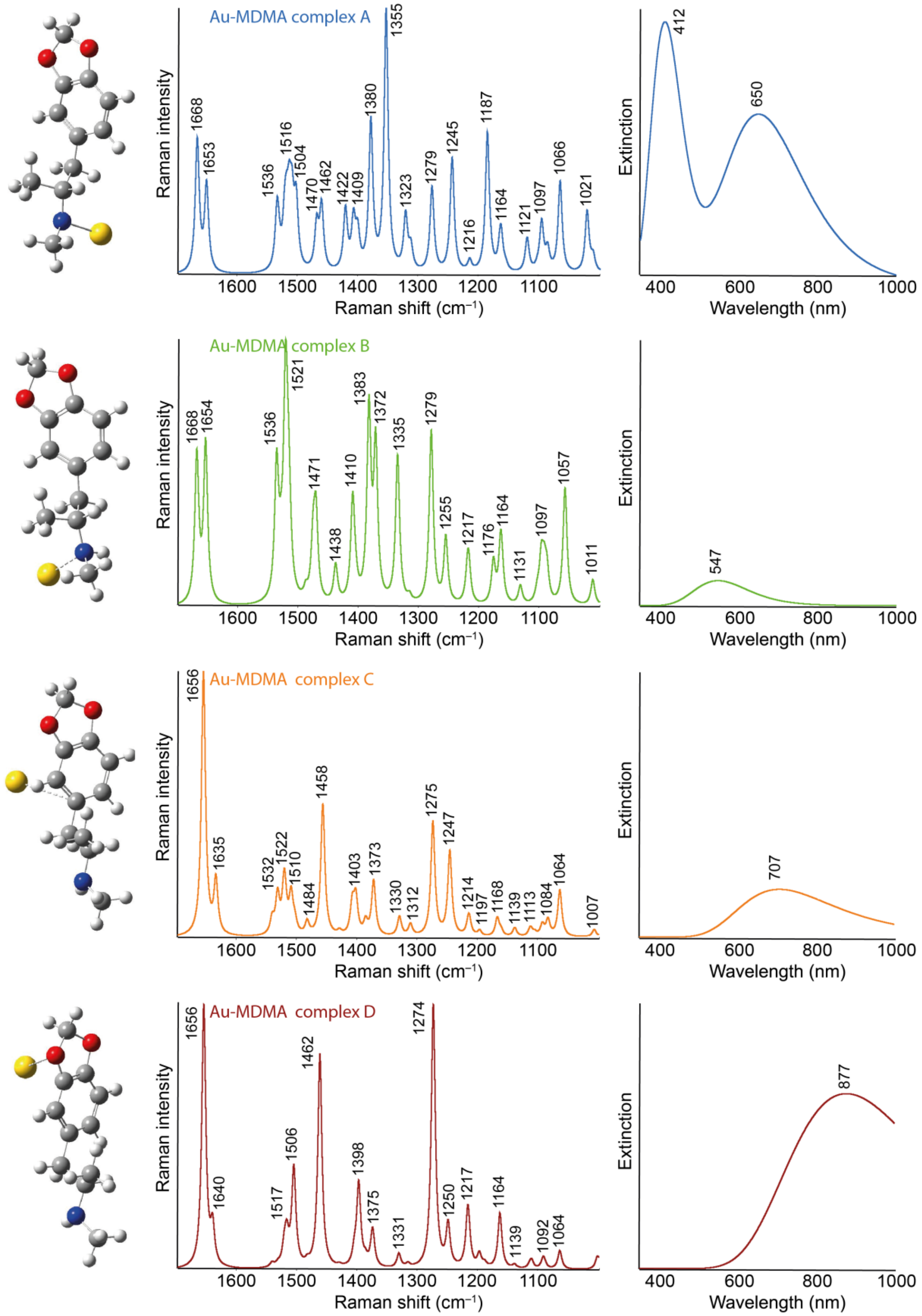

Figure S5. Structure, DFT Raman and UV-Vis spectra of the considered Au-MDMA complexes.

Table S17. Assignment of the vibrational modes of DFT spectra of Au–MDMA complex A

Raman shift (cm^{-1})		
DFT	DFT (scaled 0.98)	Assignment of the vibrational modes
1668	1635	ν $(C–C)_{ar}$
1653	1620	ν $(C=C)_{ar}$
1536	1505	δ_{sci} $(–CH_2)_O$
1516	1485	δ_{sci} $(–CH_3)_N$, δ_{sci} $(–CH_2)$
1504	1474	δ $(–CH_3)_N$, δ $(–CH_3)_C$
1470	1441	δ_{wag} $(–CH_2)_O$, δ_{ip} $(–CH)_{ar}$, δ_{twi} $(–CH_2)$
1462	1433	δ_{umb} $(–CH_3)_N$
1422	1394	δ_{umb} $(–CH_3)_C$
1409	1381	δ_{wag} $(–CH_2)_O$
1402	1374	δ_{twi} $(–CH_2)$, ν $(C–C)_{ar}$, ν $(C=C)_{ar}$, δ_{wag} $(–CH_2)_O$, δ (–CH)
1380	1353	δ_{wag} $(–CH_2)$, δ (–CH)
1355	1328	δ_{wag} $(–CH_2)$, δ (–CH)
1323	1296	δ_{wag} $(–CH_2)$, δ (–CH)
1279	1253	δ $(–CH_2)$, δ (–CH), δ $(–CH)_{ar}$
1245	1220	δ_{twi} $(–CH_2)$, δ $(–CH)_{ar}$, δ $(–CH_3)_{N,C}$
1216	1192	δ_{wag} $(–CH_2)$, δ_{wag} $(–CH_2)_O$, δ $(–CH)_{ar}$
1187	1163	ν (C–N), δ $(–CH_3)_N$
1164	1141	δ_{twi} $(–CH_2)_O$
1121	1098	δ $(–CH_3)_N$
1097	1075	skeletal vibration
1087	1065	skeletal vibration
1066	1045	δ $(–CH_3)_{aryl}$
1021	1001	δ $(–CH_3)_{aryl}$

ν – stretching, δ – deformation, sci – scissoring, twi – twisting, wag – wagging, roc – rocking vibration, ar – aromatic, ip – in-plane, oop – out of-plane

Table S18. Assignment of the vibrational modes of DFT spectra of Au–MDMA complex B

Raman shift (cm^{-1})		
DFT	DFT (scaled 0.98)	Assignment of the vibrational modes
1668	1635	ν (C–C)$_{ar}$
1654	1621	ν (C=C)$_{ar}$
1536	1505	δ_{sci} (–CH_2)$_O$
1521	1491	δ_{sci} (–CH_2)$_O$, δ_{sci} (–CH_2), δ (–CH_3)$_{N,C}$
1475	1446	δ_{umb} (–CH_3)$_N$
1471	1441	δ_{wag} (–CH_2)$_O$, δ (–CH)$_{ar}$, δ_{twi} (–CH_2), δ (–NH)
1438	1049	δ_{umb} (–CH_3)$_C$
1410	1381	δ_{wag} (–CH_2)$_O$
1383	1355	δ_{wag} (–CH_2), δ (–CH)
1372	1344	δ_{twi} (–CH_2), δ (–CH), δ (–NH)
1335	1308	δ_{wag} (–CH_2), δ (–CH)
1279	1254	skeletal vibration
1255	1230	δ (–CH_2), δ (–CH), δ (–CH)$_{ar}$
1217	1193	δ_{twi} (–CH_2), δ (–CH)$_{ar}$, δ (–CH), δ (–NH), δ (–CH_3)$_{N,C}$
1176	1152	ν (C–N), δ (–CH_3)$_{N,C}$
1164	1141	δ_{twi} (–CH_2)$_O$, δ (–CH)$_{ar}$
1131	1109	δ (–CH_3)$_{aryl}$
1097	1075	skeletal vibration
1057	1036	δ (–CH_3)$_{aryl}$
1011	991	ν (C–O), δ (–CH)$_{ar}$

ν – stretching, δ – deformation, sci – scissoring, twi – twisting, wag – wagging, roc – rocking vibration, ar – aromatic, ip – in-plane, oop – out of-plane

Table S19. Assignment of the vibrational modes of DFT spectra of Au–MDMA complex C

Raman shift (cm^{-1})		
DFT	DFT (scaled 0.98)	Assignment of the vibrational modes
1656	1622	ν (C–C)$_{ar}$
1635	1602	ν (C=C)$_{ar}$
1542	1511	δ$_{sci}$ (–CH$_3$)$_N$, δ$_{ip}$ (–NH)
1532	1502	δ$_{sci}$ (–CH$_2$)$_O$, δ$_{sci}$ (–CH$_3$)$_{N,C}$, δ$_{sci}$ (–CH$_2$)
1522	1492	δ$_{sci}$ (–CH$_2$), δ (–CH$_3$)$_{N,C}$, δ (–NH)
1510	1481	δ$_{sci}$ (–CH$_2$), δ (–CH$_3$)$_{N,C}$, δ (–NH)
1484	1454	δ$_{umb}$ (–CH$_3$)$_N$
1458	1428	ν (C–C)$_{ar}$, ν (C=C)$_{ar}$, δ$_{wag}$ (–CH$_2$)$_O$, δ (–CH)$_{ar}$
1430	1401	δ$_{umb}$ (–CH$_3$)$_C$
1409	1380	δ$_{wag}$ (–CH$_2$)$_O$, δ$_{twi}$ (–CH$_2$)
1403	1375	ν (C–C)$_{ar}$, ν (C=C)$_{ar}$, δ$_{wag}$ (–CH$_2$)$_O$, δ$_{twi}$ (–CH$_2$)
1386	1359	δ (–CH$_3$)$_{N,C}$, δ (–NH), δ (–CH), δ$_{wag}$ (–CH$_2$)
1373	1346	δ (–CH$_3$)$_{N,C}$, δ (–NH), δ (–CH), δ$_{wag}$ (–CH$_2$)
1330	1304	δ$_{wag}$ (–CH$_2$), δ (–CH)
1312	1286	δ$_{ip}$ (–CH)$_{ar}$
1275	1249	δ (–CH), δ$_{twi}$ (–CH$_2$), δ (–CH)$_{ar}$
1247	1222	δ (–CH$_3$)$_C$, δ$_{twi}$ (–CH$_2$), δ (–CH)
1214	1190	δ (–CH$_3$)$_{N,C}$, δ (–NH), δ (–CH), ν (C–C), δ (–CH)$_{ar}$
1197	1173	ν (C–N), δ (–CH$_3$)$_{N,C}$, δ$_{twi}$ (–CH$_2$)
1168	1145	δ$_{twi}$ (–CH$_2$)$_O$
1139	1116	δ (–CH$_3$)$_{aryl}$
1113	1091	skeletal vibration
1093	1071	skeletal vibration
1084	1062	skeletal vibration
1064	1043	δ (–CH$_3$)$_{aryl}$
1007	987	ν (C–O), δ (–CH)$_{ar}$

ν – stretching, δ – deformation, sci – scissoring, twi – twisting, wag – wagging, roc – rocking vibration, ar – aromatic, ip – in-plane, oop – out of-plane

Table S20. Assignment of the vibrational modes of DFT spectra of Au–MDMA complex D

Raman shift (cm^{-1})		
DFT	DFT (scaled 0.98)	Assignment of the vibrational modes
1655	1622	ν (C–C)$_{ar}$
1640	1608	ν (C=C)$_{ar}$
1517	1487	δ_{sci} (–CH_2)$_O$, δ_{sci} (–CH_2)$_{ar}$, δ_{ip} (–CH)$_{ar}$
1506	1475	δ_{sci} (–CH_2)$_O$, δ_{ip} (–CH)$_{ar}$
1462	1433	δ_{sci} (–CH_2)$_O$, δ_{ip} (–CH)$_{ar}$, δ_{twi} (–CH_2)$_{ar}$, ν (C=C)$_{ar}$
1398	1370	δ_{wag} (–CH_2)$_O$, δ_{ip} (–CH)$_{ar}$, δ_{twi} (–CH_2)$_{ar}$, ν (C–C)$_{ar}$
1375	1347	δ (–CH_2)$_{ar}$, δ (–CH)
1274	1249	ν (C–O), ν (C–C)$_{ar}$, ν (C=C)$_{ar}$, δ (–CH_2)$_{ar}$
1217	1193	δ_{twi} (–CH_2)$_O$, δ_{ip} (–CH)$_{ar}$
1139	1117	δ_{ip} (–CH)$_{ar}$
1092	1070	skeletal vibration
1064	1043	δ (–CH_3)$_N$, δ (–CH_3)$_C$, δ (–NH)

ν – stretching, δ – deformation, sci – scissoring, twi – twisting, wag – wagging, roc – rocking vibration, ar – aromatic, ip – in-plane, oop – out of-plane

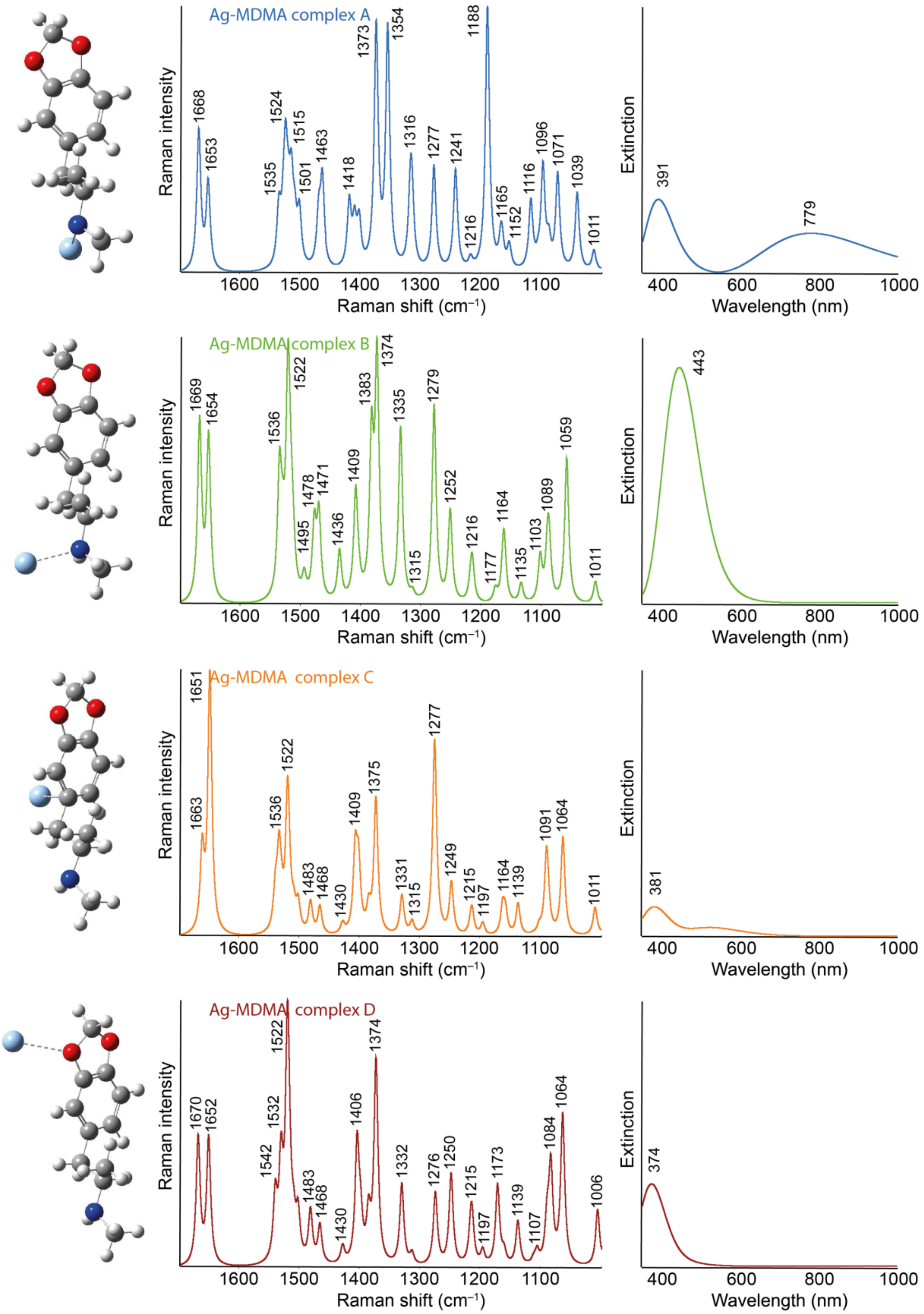

Figure S6. Structure, DFT Raman and UV-Vis spectra of the considered Ag-MDMA complexes.

Table S21. Assignment of the vibrational modes of DFT spectra of Ag–MDMA complex A

Raman shift (cm^{-1})		
DFT	DFT (scaled 0.98)	Assignment of the vibrational modes
1668	1635	ν $(C–C)_{ar}$
1653	1620	ν $(C=C)_{ar}$
1536	1505	δ_{sci} $(–CH_2)_O$
1516	1485	δ_{sci} $(–CH_3)_N$, δ_{sci} $(–CH_2)$
1504	1474	δ $(–CH_3)_N$, δ $(–CH_3)_C$
1470	1441	δ_{wag} $(–CH_2)_O$, δ_{ip} $(–CH)_{ar}$, δ_{twi} $(–CH_2)$
1462	1433	δ_{umb} $(–CH_3)_N$
1422	1394	δ_{umb} $(–CH_3)_C$
1409	1381	δ_{wag} $(–CH_2)_O$
1402	1374	δ_{twi} $(–CH_2)$, ν $(C–C)_{ar}$, ν $(C=C)_{ar}$, δ_{wag} $(–CH_2)_O$, δ (–CH)
1380	1353	δ_{wag} $(–CH_2)$, δ (–CH)
1355	1328	δ_{wag} $(–CH_2)$, δ (–CH)
1323	1296	δ_{wag} $(–CH_2)$, δ (–CH)
1279	1253	δ $(–CH_2)$, δ (–CH), δ $(–CH)_{ar}$
1245	1220	δ_{twi} $(–CH_2)$, δ $(–CH)_{ar}$, δ $(–CH_3)_{N,C}$
1216	1192	δ_{wag} $(–CH_2)$, δ_{wag} $(–CH_2)_O$ δ $(–CH)_{ar}$
1187	1163	ν (C–N), δ $(–CH_3)_N$
1164	1141	δ_{twi} $(–CH_2)_O$
1121	1098	δ $(–CH_3)_N$
1097	1075	skeletal vibration
1087	1065	skeletal vibration
1066	1045	δ $(–CH_3)_{aryl}$
1021	1001	δ $(–CH_3)_{aryl}$

ν – stretching, δ – deformation, sci – scissoring, twi – twisting, wag – wagging, roc – rocking vibration, ar – aromatic, ip – in-plane, oop – out of-plane

Table S22. Assignment of the vibrational modes of DFT spectra of Ag–MDMA complex B

Raman shift (cm^{-1})		
DFT	DFT (scaled 0.98)	Assignment of the vibrational modes
1668	1635	ν (C–C)$_{ar}$
1654	1621	ν (C=C)$_{ar}$
1536	1505	δ_{sci} (–CH_2)$_O$
1521	1491	δ_{sci} (–CH_2)$_O$, δ_{sci} (–CH_2), δ (–CH_3)$_{N,C}$
1475	1446	δ_{umb} (–CH_3)$_N$
1471	1441	δ_{wag} (–CH_2)$_O$, δ (–CH)$_{ar}$, δ_{twi} (–CH_2), δ (–NH)
1438	1049	δ_{umb} (–CH_3)$_C$
1410	1381	δ_{wag} (–CH_2)$_O$
1383	1355	δ_{wag} (–CH_2), δ (–CH)
1372	1344	δ_{twi} (–CH_2), δ (–CH), δ (–NH)
1335	1308	δ_{wag} (–CH_2), δ (–CH)
1279	1254	skeletal vibration
1255	1230	δ (–CH_2), δ (–CH), δ (–CH)$_{ar}$
1217	1193	δ_{twi} (–CH_2), δ (–CH)$_{ar}$, δ (–CH), δ (–NH), δ (–CH_3)$_{N,C}$
1176	1152	ν (C–N), δ (–CH_3)$_{N,C}$
1164	1141	δ_{twi} (–CH_2)$_O$, δ (–CH)$_{ar}$
1131	1109	δ (–CH_3)$_{aryl}$
1097	1075	skeletal vibration
1057	1036	δ (–CH_3)$_{aryl}$
1011	991	ν (C–O), δ (–CH)$_{ar}$

ν – stretching, δ – deformation, sci – scissoring, twi – twisting, wag – wagging, roc – rocking vibration, ar – aromatic, ip – in-plane, oop – out of-plane

Table S23. Assignment of the vibrational modes of DFT spectra of Ag–MDMA complex C

Raman shift (cm^{-1})		
DFT	DFT (scaled 0.98)	Assignment of the vibrational modes
1656	1622	ν (C–C)$_{ar}$
1635	1602	ν (C=C)$_{ar}$
1542	1511	δ_{sci} (–CH_3)$_N$, δ_{ip} (–NH)
1532	1502	δ_{sci} (–CH_2)$_O$, δ_{sci} (–CH_3)$_{N,C}$, δ_{sci} (–CH_2)
1522	1492	δ_{sci} (–CH_2), δ (–CH_3)$_{N,C}$, δ (–NH)
1510	1481	δ_{sci} (–CH_2), δ (–CH_3)$_{N,C}$, δ (–NH)
1484	1454	δ_{umb} (–CH_3)$_N$
1458	1428	ν (C–C)$_{ar}$, ν (C=C)$_{ar}$, δ_{wag} (–CH_2)$_O$, δ (–CH)$_{ar}$
1430	1401	δ_{umb} (–CH_3)$_C$
1409	1380	δ_{wag} (–CH_2)$_O$, δ_{twi} (–CH_2)
1403	1375	ν (C–C)$_{ar}$, ν (C=C)$_{ar}$, δ_{wag} (–CH_2)$_O$, δ_{twi} (–CH_2)
1386	1359	δ (–CH_3)$_{N,C}$, δ (–NH), δ (–CH), δ_{wag} (–CH_2)
1373	1346	δ (–CH_3)$_{N,C}$, δ (–NH), δ (–CH), δ_{wag} (–CH_2)
1330	1304	δ_{wag} (–CH_2), δ (–CH)
1312	1286	δ_{ip} (–CH)$_{ar}$
1275	1249	δ (–CH), δ_{twi} (–CH_2), δ (–CH)$_{ar}$
1247	1222	δ (–CH_3)$_C$, δ_{twi} (–CH_2), δ (–CH)
1214	1190	δ (–CH_3)$_{N,C}$, δ (–NH), δ (–CH), ν (C–C), δ (–CH)$_{ar}$
1197	1173	ν (C–N), δ (–CH_3)$_{N,C}$, δ_{twi} (–CH_2)
1168	1145	δ_{twi} (–CH_2)$_O$
1139	1116	δ (–CH_3)$_{aryl}$
1113	1091	skeletal vibration
1093	1071	skeletal vibration
1084	1062	skeletal vibration
1064	1043	δ (–CH_3)$_{aryl}$
1007	987	ν (C–O), δ (–CH)$_{ar}$

ν – stretching, δ – deformation, sci – scissoring, twi – twisting, wag – wagging, roc – rocking vibration, ar – aromatic, ip – in-plane, oop – out of-plane

Table S24. Assignment of the vibrational modes of DFT spectra of Ag–MDMA complex D

Raman shift (cm^{-1})		
DFT	DFT (scaled 0.98)	Assignment of the vibrational modes
1655	1622	ν (C–C)$_{ar}$
1640	1608	ν (C=C)$_{ar}$
1517	1487	δ_{sci} (–CH$_2$)$_O$, δ_{sci} (–CH$_2$)$_{ar}$, δ_{ip} (–CH)$_{ar}$
1506	1475	δ_{sci} (–CH$_2$)$_O$, δ_{ip} (–CH)$_{ar}$
1462	1433	δ_{sci} (–CH$_2$)$_O$, δ_{ip} (–CH)$_{ar}$, δ_{twi} (–CH$_2$)$_{ar}$, ν (C=C)$_{ar}$
1398	1370	δ_{wag} (–CH$_2$)$_O$, δ_{ip} (–CH)$_{ar}$, δ_{twi} (–CH$_2$)$_{ar}$, ν (C–C)$_{ar}$
1375	1347	δ (–CH$_2$)$_{ar}$, δ (–CH)
1274	1249	ν (C–O), ν (C–C)$_{ar}$, ν (C=C)$_{ar}$, δ (–CH$_2$)$_{ar}$
1217	1193	δ_{twi} (–CH$_2$)$_O$, δ_{ip} (–CH)$_{ar}$
1139	1117	δ_{ip} (–CH)$_{ar}$
1092	1070	skeletal vibration
1064	1043	δ (–CH$_3$)$_N$, δ (–CH$_3$)$_C$, δ (–NH)

ν – stretching, δ – deformation, sci – scissoring, twi – twisting, wag – wagging, roc – rocking vibration, ar – aromatic, ip – in-plane, oop – out of-plane

S5. Narrow Interval Chemical Enhancement

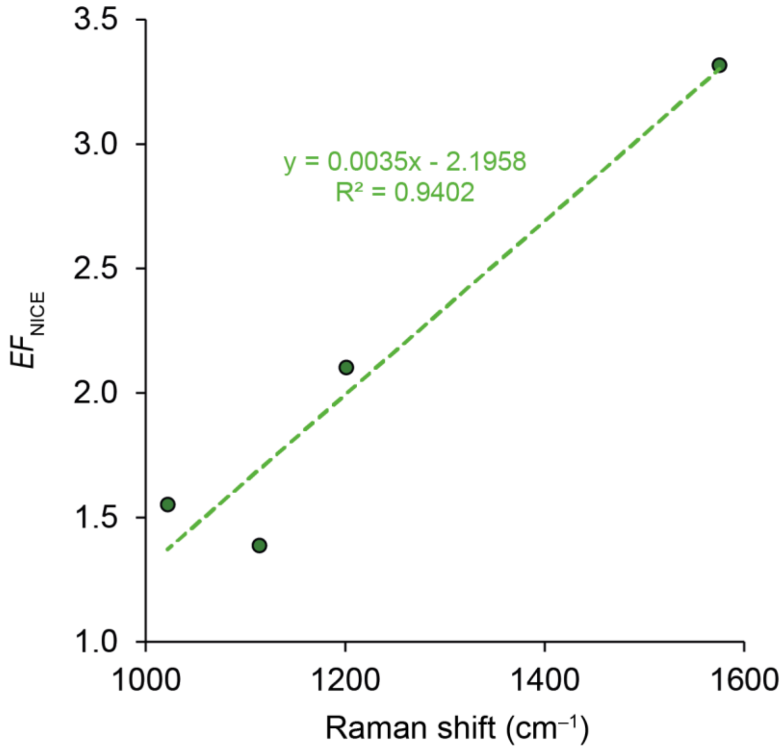

Figure S7. NICE enhancement factor values for AMP-modified AuNPs.

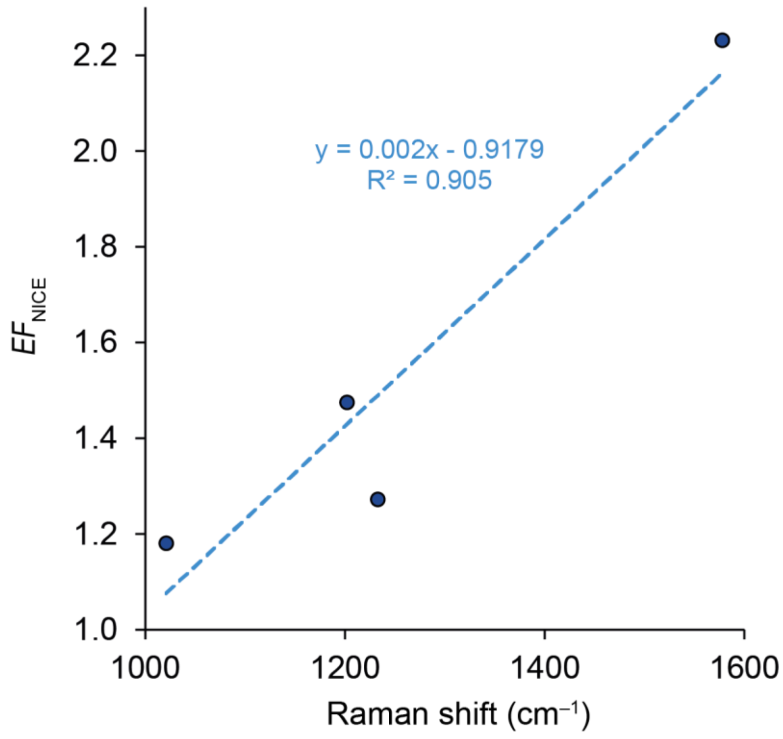

Figure S8. NICE enhancement factor values for MET-modified AuNPs.

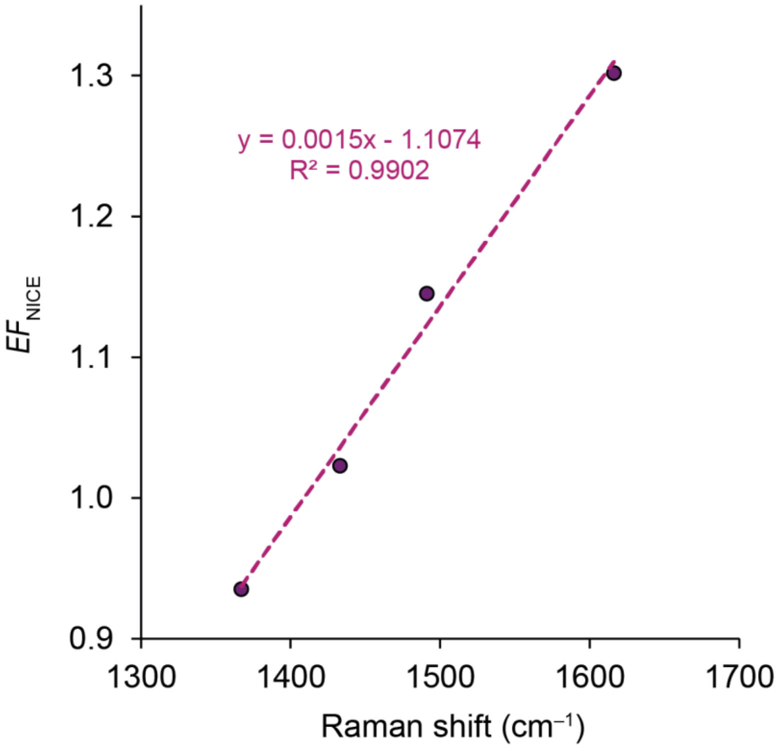

Figure S9. NICE enhancement factor values for MDMA-modified AuNPs.

S6. Excitation wavelength dependence

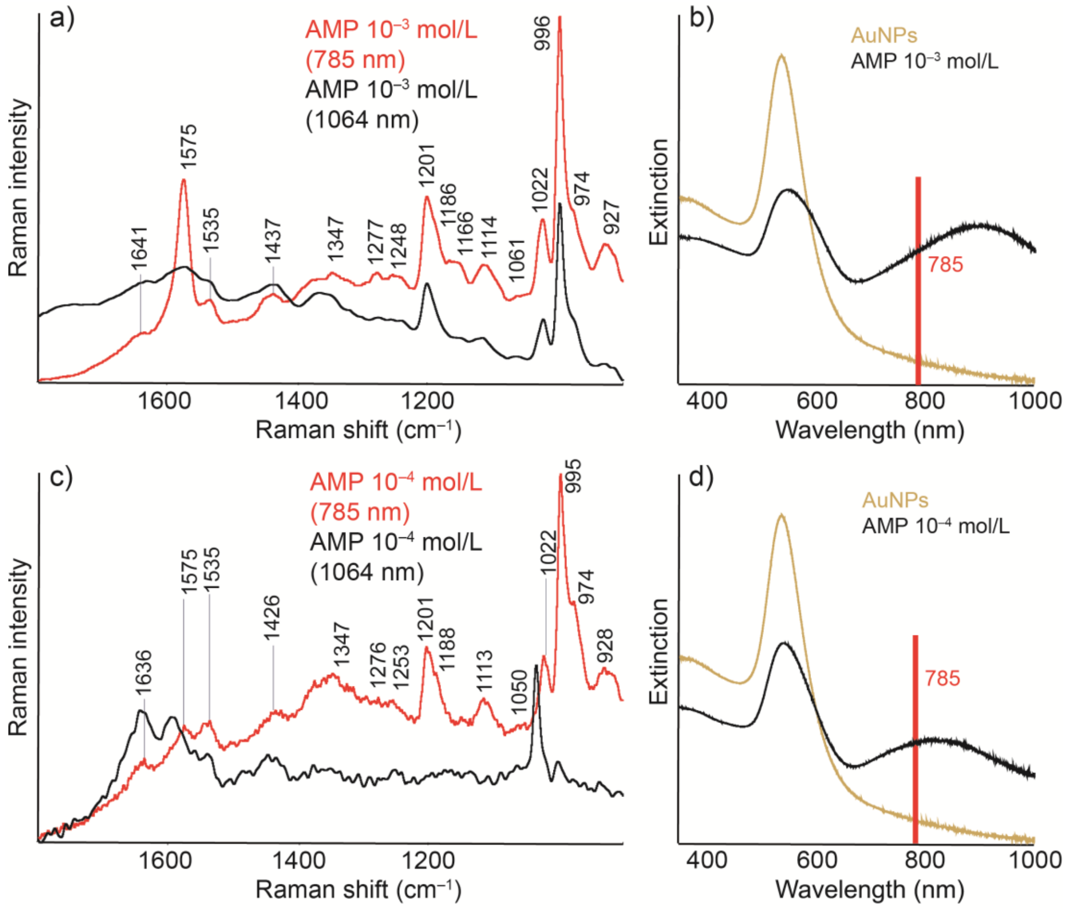

Figure S10. Experimental Au-SERS (a,c) and extinction (b,d) spectra of AuNPs modified by MET of concentration 10^{-3} mol/L (a, b) and 10^{-4} mol/L (c, d). SERS spectra measured with different wavelengths do not share the same scale for better readability.

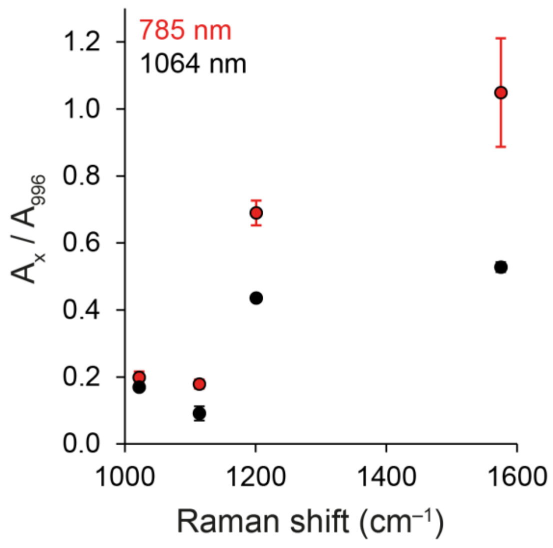

Figure S11. Comparison of excitation wavelength-dependent trends of SERS area ratios of selected AMP Au-SERS bands.

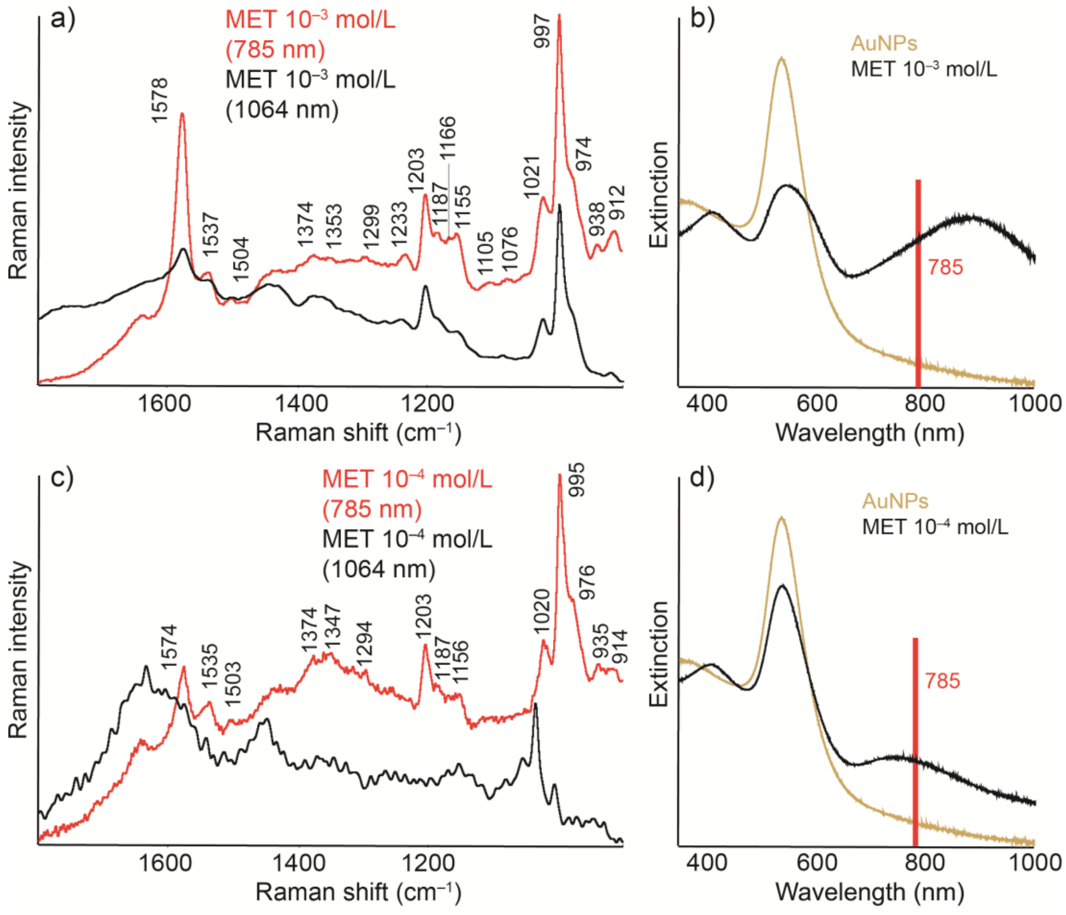

Figure S12. Experimental Au-SERS (a,c) and extinction (b,d) spectra of AuNPs modified by MET of concentration 10^{-3} mol/L (a, b) and 10^{-4} mol/L (c, d). SERS spectra measured with different wavelengths do not share the same scale for better readability.

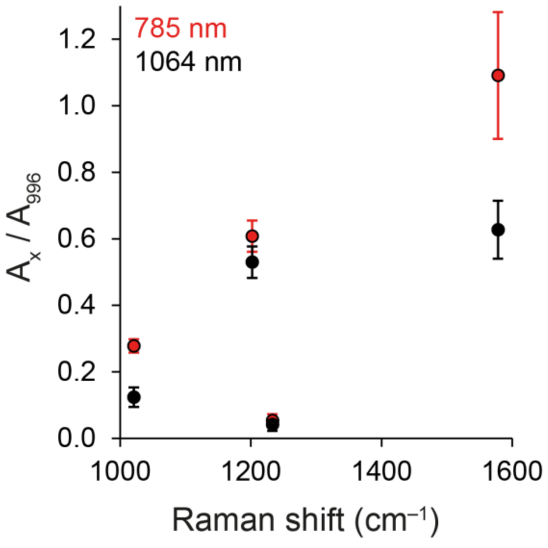

Figure S13. Comparison of excitation wavelength-dependent trends of SERS area ratios of selected MET Au-SERS bands.

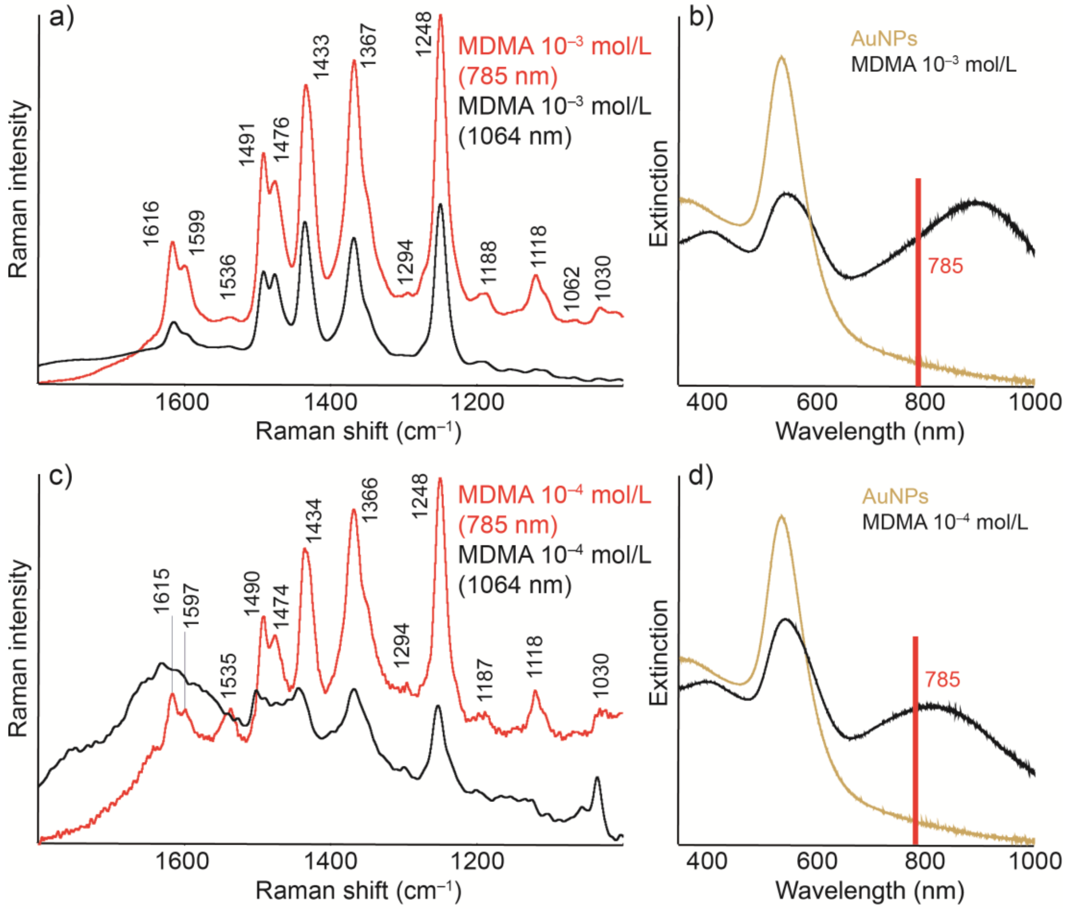

Figure S14. Experimental Au-SERS (a,c) and extinction (b,d) spectra of AuNPs modified by MDMA of concentration 10^{-3} mol/L (a, b) and 10^{-4} mol/L (c, d). SERS spectra measured with different wavelengths do not share the same scale for better readability.

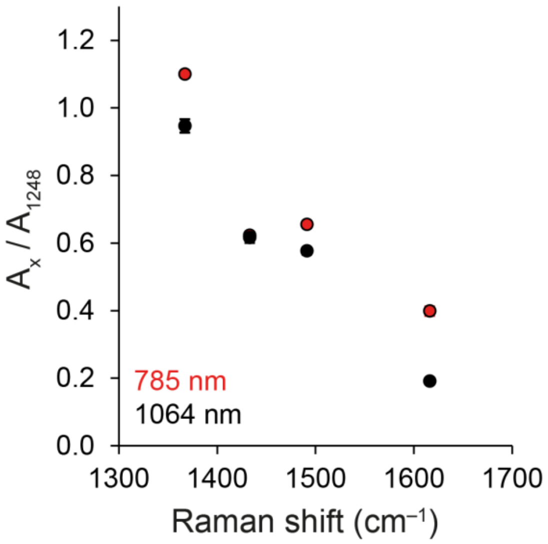

Figure S15. Comparison of excitation wavelength-dependent trends of SERS area ratios of selected MDMA Au-SERS bands.

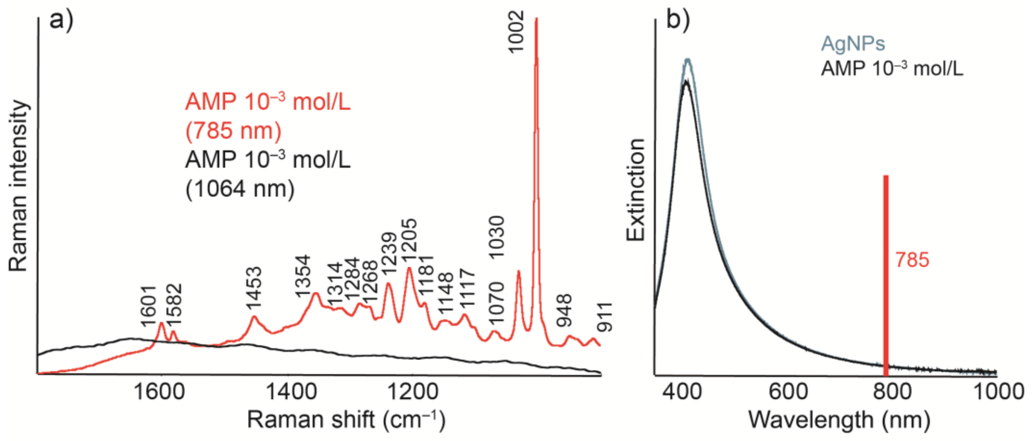

Figure S16 Experimental Ag-SERS (a) and extinction (b) spectra of AgNPs modified by AMP of concentration 10^{-3} mol/L. SERS spectra measured with different wavelengths do not share the same scale for better readability.

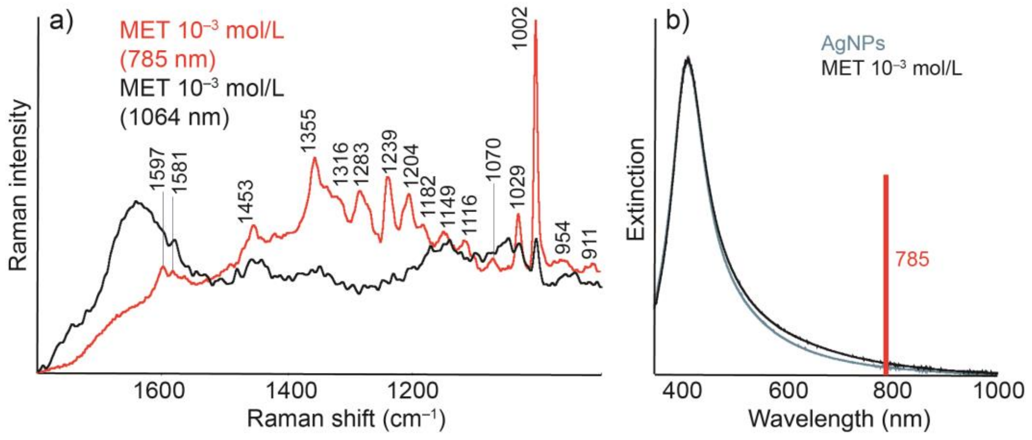

Figure S17. Experimental Ag-SERS (a) and extinction (b) spectra of AgNPs modified by MET of concentration 10^{-3} mol/L. SERS spectra measured with different wavelengths do not share the same scale for better readability.

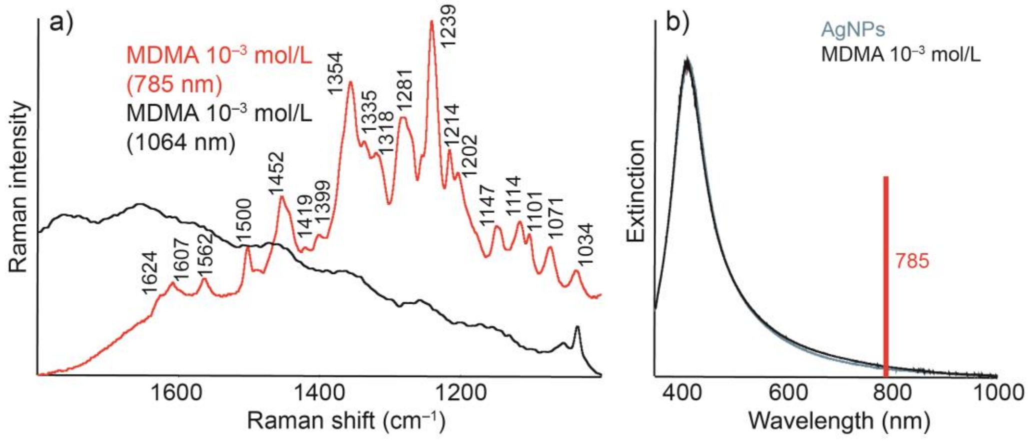

Figure S18. Experimental Ag-SERS (a) and extinction (b) spectra of AgNPs modified by MDMA of concentration 10^{-3} mol/L. SERS spectra measured with different wavelengths do not share the same scale for better readability.

REFERENCES

(1) Tódor, I. S.; Szabó, L.; Marişca, O. T.; Chiş, V.; Leopold, N. Gold nanoparticle assemblies of controllable size obtained by hydroxylamine reduction at room temperature. *J. Nanopart. Res*. **2014**, *16* (12), 2740. doi.org/10.1007/s11051-014-2740-4.

(2) Leopold, N.; Lendl, B. A New Method for Fast Preparation of Highly Surface-Enhanced Raman Scattering (SERS) Active Silver Colloids at Room Temperature by Reduction of Silver Nitrate with Hydroxylamine Hydrochloride. *J. Phys. Chem. B.* **2003**, *107* (24), 5723-5727. doi.org/10.1021/jp027460u.
